# A protein disulfide isomerase coordinates redox homeostasis and ER calcium regulation for optimal lytic cycle progression in *Toxoplasma gondii*

**DOI:** 10.1128/mbio.03124-25

**Published:** 2026-04-01

**Authors:** Katherine E. Moen, Silvia N. J. Moreno

**Affiliations:** 1 Center for Tropical and Emerging Global Diseases, University of Georgia 1355, Athens, Georgia, USA; 2 Department of Cellular Biology, University of Georgia 1355, Athens, Georgia, USA; The University of Arizona, Tucson, Arizona, USA

**Keywords:** *Toxoplasma gondii*, endoplasmic reticulum, protein disulfide isomerase, calcium homeostasis, SERCA, redox regulation

## Abstract

The endoplasmic reticulum (ER) maintains an oxidative environment that facilitates the formation of disulfide bonds, a critical process for proper protein folding. Protein disulfide isomerases (PDIs) are ER-resident enzymes that facilitate the formation, breakage, and rearrangement of disulfide bonds between cysteine residues, thereby stabilizing protein structures. Although PDIs are functionally diverse, they all contain at least 1 thioredoxin-like domain and most mediate disulfide exchange through their conserved CXXC motifs. The apicomplexan parasite, *Toxoplasma gondii*, infects approximately one-third of the world population, posing a significant risk to immunosuppressed individuals and unborn fetuses. The fast-replicating tachyzoite form engages in a lytic cycle, causing host tissue damage and contributing to pathogenesis. While approximately 26 PDIs are predicted to be present in *T. gondii*, their specific roles remain largely unexplored. In this study, we investigate TgPDIA3, a *T. gondii* PDI localized to the ER, along with several of its interacting protein substrates. We explore its role in ER redox activity and calcium sequestration and assess how these functions contribute to the parasite’s lytic cycle.

**IMPORTANCE:**

The lytic cycle of *Toxoplasma gondii* is critical for parasite dissemination and disease progression in the host. Calcium signaling plays a crucial role in driving these processes; however, the molecules that control calcium storage and release remain poorly understood. The endoplasmic reticulum, likely the largest calcium reservoir in *T. gondii*, has been understudied in the context of calcium signaling. Here, we uncover a direct link between ER redox regulation and calcium homeostasis, showing that ER redox activity can influence calcium signaling events that govern microneme protein maturation and secretion, parasite invasion, and replication. Our findings indicate that redox-dependent calcium regulation in the ER contributes to control of the parasite lytic cycle and reveals a previously unrecognized mechanism that may influence parasite virulence.

## INTRODUCTION

*Toxoplasma gondii* is an obligate intracellular apicomplexan parasite that infects approximately one-third of the world’s human population (1). In the United States, there is an estimated seroprevalence of 11% within individuals 6 years and older (2). Reactivation of *T. gondii* latent infections in individuals who become immunocompromised can cause toxoplasmic encephalitis (3, 4). Congenital transmission can occur when women become newly infected during pregnancy (5). During the acute infection, the fast-growing tachyzoite engages in a lytic cycle, during which it attaches and actively invades host cells and undergoes asexual replication inside a parasitophorous vacuole (PV), followed by egress, rupturing the host cell’s membranes. This lytic cycle drives the parasite’s pathogenesis by continuously disrupting host tissues and spreading through all host organs (6, 7). *T. gondii* possesses specialized organelles called micronemes and rhoptries, unique to apicomplexans, that secrete microneme and rhoptry proteins, respectively. Microneme proteins are secreted during egress, gliding, host cell attachment, and invasion (8). Following attachment, rhoptry neck (RON) and rhoptry bulb (ROP) proteins are released to facilitate parasite entry into the host cell and contribute to the development of the PV (9). Elevation of cytosolic calcium (Ca^2+^) through treatment with ionophores triggers microneme secretion, a crucial event for most transitions in the parasites’ lytic cycle (10).

Calcium signaling is universal and regulates a wide range of processes in all eukaryotic cells (11, 12). However, cells must maintain a low resting level of cytosolic Ca^2+^ (<100 nM) because sustained elevations are cytotoxic and can disrupt cellular processes. Cells sequester Ca^2+^ in intracellular stores, particularly the endoplasmic reticulum (ER), which serves as the largest intracellular Ca^2+^ store in most eukaryotic cells (12). ER calcium homeostasis is maintained by calcium-binding proteins (CBPs) within the ER lumen, in addition to calcium channels, pumps, and exchangers located on the ER membrane. The sarco-endoplasmic reticulum Ca^2+^-ATPase (SERCA) pumps Ca^2+^ into the ER and helps maintain low cytosolic levels. In mammals, the inositol trisphosphate receptor (IP_3_R) and the ryanodine receptor (RyR) are channels embedded in the ER membrane that, when activated, allow Ca^2+^ release into the cytosol to initiate signaling (11).

The high concentration of Ca^2+^ in the ER is essential for its multiple functions, including signaling, protein chaperoning, and maintaining redox homeostasis. The ER serves as the cell’s quality control center for post-translational processing of membrane proteins (13) and proteins destined for secretion (14). Molecular chaperones use hydrophobic interactions, often coupled to ATP hydrolysis, to fold nascent polypeptides into their correct three-dimensional conformations (15). Binding immunoglobulin protein (BiP), an ER resident HSP70 molecular chaperone, binds Ca^2+^ for sensing and storage, and its chaperone activity is regulated by calcium binding (16, 17). Lectin chaperones in the ER play a crucial role in folding glycoproteins and often function as low-affinity, high-capacity CBPs, that are important for calcium sensing and storage. Calcium binding also modulates their chaperone functions (17).

**Fig 1:**
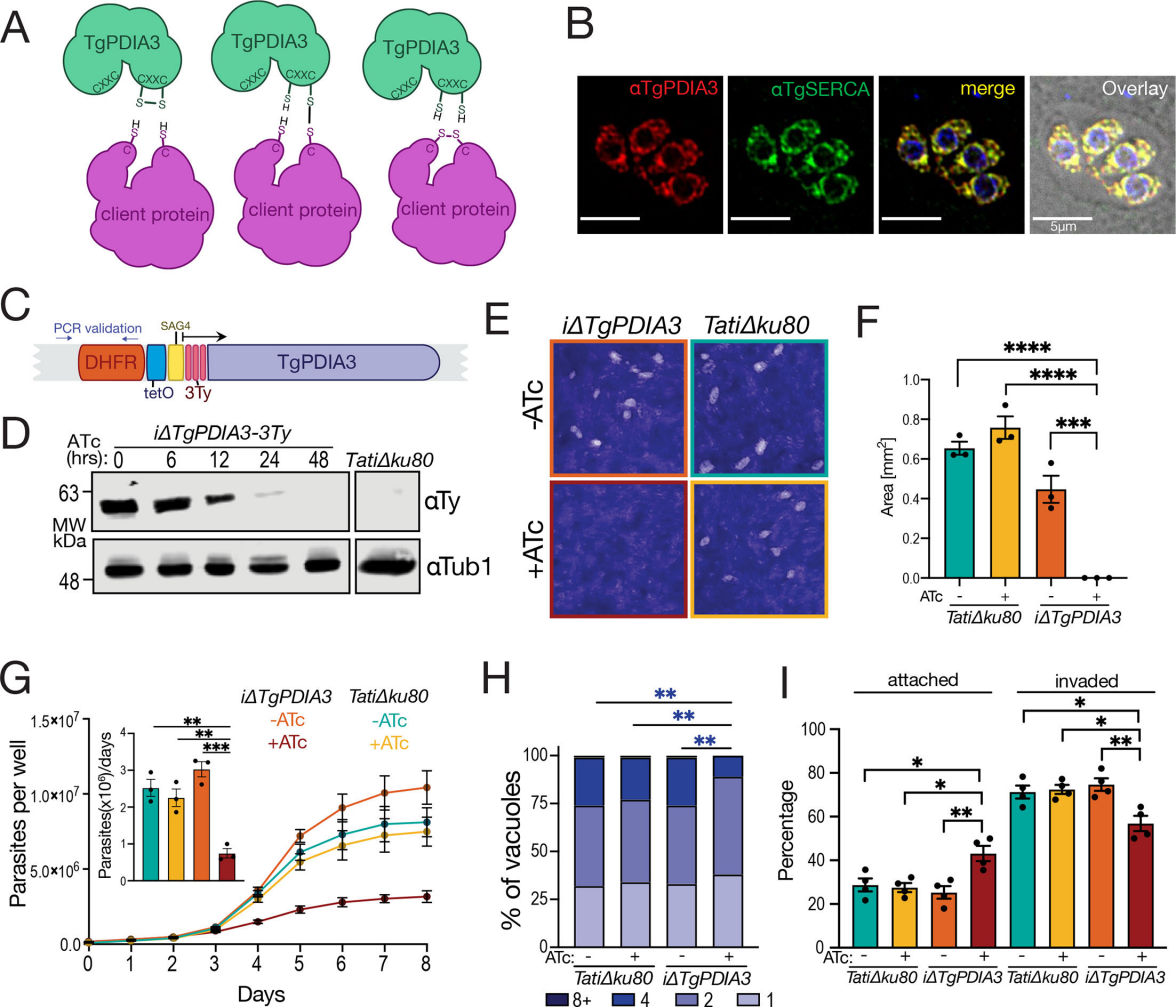
TgPDIA3 is an essential ER-resident protein . (**A**) Scheme showing the redox activity of TgPDIA3 on a client protein. (**B**) IFA of intracellular parasites using αTgPDIA3 antibody and co-localization with αTgSERCA (scale bar, 5 μm). (**C**) Scheme showing the promoter insertion strategy for the generation of the *iΔTgPDIA3-3Ty* mutant. DHFR, dihydrofolate reductase used for pyrimethamine selection, tetO operator, SAG4 promoter, 3xTy tag, and the location of primers used in PCR validation (Fig. S1F). (**D**) Western blots of *iΔTgPDIA3-3Ty* or *TatiΔku80* lysates probed with αTy and αTubulin (control) in parasites grown in the presence of ATc for the indicated times. (**E**) plaque assays of the *iΔTgPDIA3-3Ty* mutant and *TatiΔku80* (control) grown for 7 days ± ATc as indicated. (**F**) Quantification of plaque sizes (mm^2^) measured using ImageJ, using one-way ANOVA for statistical analysis (*n* = 3). Plaques that were not visible were considered 0 mm^2^. (**G**) Growth assay of *iΔTgPDIA3-3Ty-RFP* and *TatiΔku80-RFP* (control) parasites grown in the presence or absence of ATc. Parasite numbers were estimated from standard curves generated from red fluorescence measurements of known parasite numbers, with calibration performed separately for each mutant. The inset bar graph shows quantification of the growth rate for days 3–5 using one-way ANOVA for statistical analysis (*n* = 3). (**H**) Quantification of replication assay showing the percentage of vacuoles containing 1, 2, 4, or ≥8 parasites, 20 h post-infection. Centered log-ratio (CLR**)**-transformed data were analyzed using a linear mixed model (*n* = 4). (**I**) Quantification of parasite invasion measured using a red-green assay as the percentage of parasites invaded or attached vs. total parasites. Data were analyzed using one-way ANOVA (*n* = 4). ****P < 0.0001; ***P < 0.001; ** *P* < 0.01; *P < 0.05; ns, *P* ≥ 0.05.

The ER is an oxidizing environment (18) and, as such, serves as a major site of oxidative protein folding within the cell. This post-translational process, carried out by ER redox-active enzymes, consists of the formation, cleavage, and rearrangement of disulfide bonds in client proteins, stabilizing them in their correct conformations. Among these enzymes are protein disulfide isomerases (PDIs), which belong to the thioredoxin (TRX) superfamily of dithiol/disulfide oxidoreductases (19–22). Redox-active PDIs contain canonical CXXC motifs, which cycle between oxidized and reduced states to catalyze the formation, cleavage, or rearrangement of disulfide bonds in their protein substrates (Fig. 1A). Humans possess 21 known PDIs, many of which are multifunctional. Most PDIs feature specialized b or b’ domains rich in hydrophobic residues that are important for substrate recognition and assist in non-redox protein folding (23–26). Some PDIs contain low-affinity, high-capacity calcium-binding domains that contribute to ER Ca^2+^ buffering and sensing (27). Additionally, some PDIs regulate other proteins through disulfide bond exchange. In vertebrates, several PDIs and other ER redox proteins play a role in calcium homeostasis by adding or removing regulatory disulfide bonds on SERCA or IP_3_R (27–30).

In the ER, chaperoning, redox, and calcium homeostasis are interconnected and delicately balanced. ER stress can be triggered by depletion of ER Ca^2+^, which lowers the folding capacity of the ER and leads to accumulation of misfolded proteins, disrupting ER redox homeostasis. Furthermore, accumulation of misfolded proteins may lead to the disruption of calcium and redox homeostasis, triggering ER stress (31, 32). Disturbances in ER redox homeostasis can also lead to the accumulation of misfolded proteins and cause ER stress (33, 34). Some redox proteins, in collaboration with other chaperones, interact with IP_3_R at mitochondria-associated membranes (MAMs), which regulates Ca^2^ transfer to the mitochondria (28, 35, 36). In this way, ER stress caused by excessive Ca^2+^ accumulation can lead to either an increase in ATP production or the initiation of apoptosis (37).

PDIs operate at the intersection of these finely balanced processes, serving as communication links among various ER functions. Nearly all PDIs are redox-active and play a central role in maintaining ER redox homeostasis. While some PDIs modulate ER Ca^2+^ levels through their interactions with ER calcium pumps and channels, others directly bind, sense, and sequester Ca^2+^ within the ER. Beyond stabilizing client proteins with disulfide bond formation, some PDIs interact with molecular and lectin chaperones, influencing and being influenced by ER chaperone activity (26, 38, 39).

The *T. gondii* genome encodes 26 predicted PDIs (Table S1). In this study, we characterize one of these proteins, a *T. gondii* PDI, predicted to be a putative ortholog to human PDIA3 (also known as ERp57 or GRP58). We investigated the enzymatic activity of this *T. gondii* PDI and its involvement in various ER functions, including the maintenance of ER calcium homeostasis, interactions with potential substrates, and association with secreted effectors. We present a functional characterization of this PDI and its substrates, highlighting their biochemical roles and resulting impact on the *T. gondii* lytic cycle.

## RESULTS

### TgPDIA3 is an essential ER resident protein

To understand how redox regulation contributes to calcium signaling and homeostasis, we focused on two *T. gondii* protein disulfide isomerases with potential links to these processes. *TGGT1_211680* is predicted to encode a PDI with a calcium-binding function, while the mammalian homolog of *TGGT1_249270* (PDIA6) has been implicated in ER protein quality control, stress signaling, and calcium buffering (20), providing the rationale for examining its *T. gondii* counterpart. The CRISPR fitness scores for both genes, as reported in a genomic screen by Sidik et al. (40), indicate that disruption of either gene results in a substantial fitness cost. We termed these proteins TgPDIA3 (TGGT1_211680) and TgPDIA6 (TGGT1_249270) with their corresponding genes named *Tgpdia3* and *Tgpdia6*. Phylogenetic analysis of TgPDIA3 (Fig. S1A and Table S2), including BLASTP comparisons with predicted orthologous proteins across different kingdoms, revealed a close evolutionary relationship with human PDIA3 and human PDIA4 sharing approximately 34% sequence identity with each other. The domain architecture of TgPDIA3 mirrors that of HsPDIA3, both featuring four globular domains, while HsPDIA4 contains five (Fig. S1B). The **a** catalytic domains of all 3 PDIs share the canonical CGHC active site (41). However, the **a′** domain of TgPDIA3 displays a substitution of histidine (H) for tyrosine (Y), replacing a charged residue with an uncharged one, which could potentially alter its redox function (Fig. S1C). Using AlphaFold predicted models (42, 43), we visualized the spatial orientation of TgPDIA3 catalytic motifs and found that both face inward toward each other (Fig. S1D and E), similar to HsPDIA3 (44, 45), suggesting potential functional similarities. A sequence BLASTP analysis revealed that the protein encoded by *TGGT1_249270* shares the highest sequence homology with HsPDIA6, with ~42% sequence identity. Structural overlays of the AlphaFold-predicted models demonstrated a high degree of structural similarity between TgPDI6 and HsPDIA6 (Fig. S2A and B).

Like many ER proteins, both TgPDIA3 and TgPDIA6 possess an N-terminal signal peptide and a C-terminal retention signal. While the most common retention motifs are KDEL or HDEL, TgPDIA3 instead features the uncommon GEEL sequence. Because of the presence of this retention signal, we predicted that adding a C-terminal tag could alter the localization of the protein, while an N-terminal tag could be cleaved off during maturation. Hence, we generated a TgPDIA3 antibody in mice to visualize endogenous TgPDIA3. The *Tgpdia3* gene was first cloned into a bacterial expressing vector, followed by expression and purification of the TgPDIA3 protein. The purified protein was used as an antigen for immunizing mice and generating αTgPDIA3 antibodies (Fig. S3A and B). The specificity of these antibodies was validated by western blots of parasite lysates (Fig. S3C). Immunofluorescence assays (IFAs) using the generated antibodies successfully visualized endogenous TgPDIA3, which localized to the ER, as confirmed by its co-localization with αTgSERCA (46), an established ER marker (Fig. 1B; Fig. S3D). To visualize TgPDIA6, we introduced a 3-copy hemagglutinin (3× HA) tag, with its KDEL retention signal downstream. ER localization of the tagged TgPDIA6 was demonstrated through co-localization with αTgSERCA (Fig. S2C).

With the aim of investigating the role of TgPDIA3 and TgPDIA6 in *T. gondii*, we generated conditional knockdown mutants (*iΔTgPDIA3-3Ty*, *iΔTgPDIA3* [no tag], and *iΔTgPDIA6-3Ty*) by inserting a tetracycline response element (47) upstream of a SAG4 promoter at the 5’ end of each gene (Fig. 1C). In this system, gene expression is downregulated by adding anhydrotetracycline (ATc) to the culture media. Successful gene tagging was confirmed by PCR (Fig. S1F and S2D), and downregulation of protein expression was validated by western blot (Fig. 1D and Fig. S2E). To evaluate the role of TgPDIA3 and TgPDIA6 in the *T. gondii* lytic cycle, we performed plaque assays, in which the parasite engages in cycles of invasion, replication, and egress, causing host cell lysis. The resulting plaques appear as cleared zones in the host cell monolayer stained with crystal violet. Parasites lacking TgPDIA3 formed no visible plaques, indicating that TgPDIA3 is important for at least one step of the lytic cycle (Fig. 1E and F). Despite the CRISPR fitness score of −4.56 predicted for TgPDIA6, suggesting a strong fitness defect, the *iΔTgPDIA6-3Ty* mutant exhibited no significant differences in plaque size compared to controls (Fig. S2F and G). To confirm these findings, we transfected both *iΔTgPDIA3* and *iΔTgPDIA6-3Ty* mutants with a cytosolic red fluorescent protein (RFP) gene and isolated *iΔTgPDIA3-RFP* and *iΔTgPDIA6-RFP* mutants. Growth assays measured red fluorescence over 8 days, and using a standard curve, we calculated parasite numbers based on fluorescence intensity. In the *iΔTgPDIA3-RFP* mutant, parasite growth rate was significantly decreased at 4 days of ATc treatment (Fig. 1G), supporting the importance of TgPDIA3 for the lytic cycle. However, the *iΔTgPDIA6-RFP* mutant showed no significant growth differences compared to controls (Fig. S2H). Further exploring the role of TgPDIA3 in the lytic cycle, we performed replication assays by allowing control and *iΔTgPDIA3-RFP* parasites to invade hTERT cells. Twenty hours post-infection (hpi), parasitophorous vacuoles containing 1, 2, 4, or 8+ parasites were enumerated. The *iΔTgPDIA3-RFP* mutant cultured with ATc exhibited a significant reduction in the number of PVs with four or eight parasites compared to controls (-ATc or *TatiΔku80*), indicating that TgPDIA3 plays a significant role in replication (Fig. 1H). To investigate the role of TgPDIA3 in host cell invasion, we conducted a modified red-green assay (48, 49). After allowing invasion, extracellular RFP-expressing parasites were probed with αSAG1 to distinguish them from the invaded parasites. The *iΔTgPDIA3-RFP* mutant pre-incubated with ATc for 48 h exhibited a significant reduction in invasion and a corresponding increase in parasite attachment compared to controls (Fig. 1I), indicating that TgPDIA3 is important for *T. gondii* host cell invasion.

**Fig 2:**
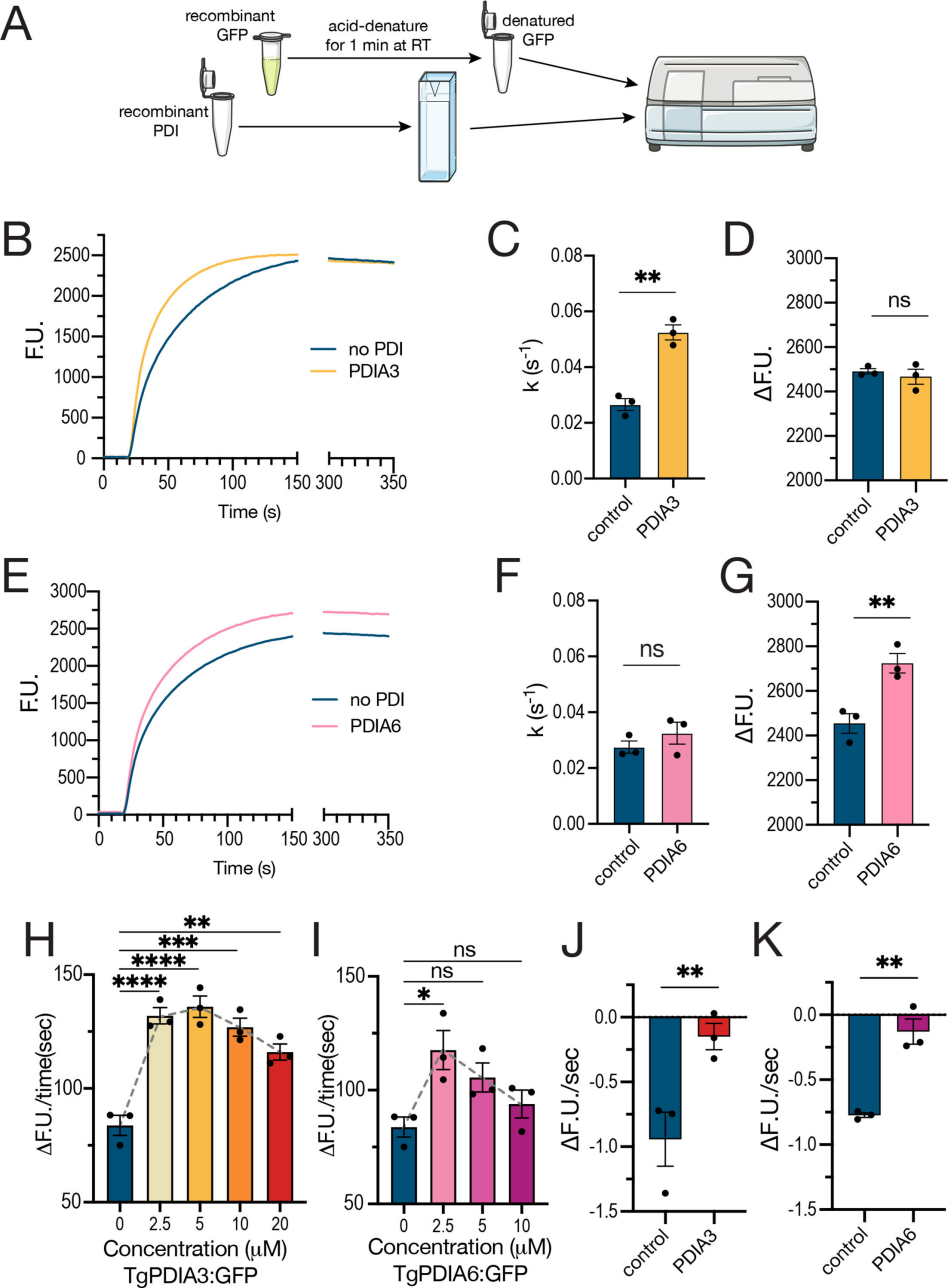
TgPDIA3 and TgPDIA6 enhance the refolding of denatured GFP *in vitro*. (**A**) Scheme of the protein refolding assay utilizing recombinant acid-denatured GFP and recombinant TgPDIA3 or TgPDIA6. (**B**) Average traces of GFP fluorescence recovery (1 µM ) in the absence of PDI (control) or in the presence of 5 µM TgPDIA3. (**C**) Rate constant (*k*) calculated by nonlinear regression (curve fit) using a one-phase association model, starting at the 20-s time point, for GFP fluorescence recovery measured in the presence of 5 µM TgPDIA3 compared to control reactions lacking PDI. (**D**) Quantification of the total change in GFP fluorescence in the presence of 5 µM TgPDIA3 or in its absence (control). (**E**) Average traces of GFP fluorescence recovery in the absence of PDI (control) or in the presence of 2.5 µM TgPDIA6. (**F**) Rate constant (*k*) of GFP fluorescence recovery measured in the presence of 2.5 µM TgPDIA6. (**G**) Quantification of total change in GFP fluorescence in the absence or presence of 2.5 µM TgPDIA6. (**H**) Quantification of the initial 7-s slope of GFP fluorescence recovery at varying concentrations of TgPDIA3. Statistical analysis was performed using one-way ANOVA (*n* = 3). (**I**) Quantification of the initial 7-s slope of GFP fluorescence recovery at varying concentrations of TgPDIA6. Statistical analysis was performed using one-way ANOVA (*n* = 3). (**J**) Quantification of the slope of GFP fluorescence loss after the initial recovery with 20 µM TgPDIA3 or in its absence (control). (**K**) Quantification of the slope of GFP fluorescence loss after the initial recovery in the presence of 10 µM TgPDIA6 or in its absence (control). Quantifications were done with Student’s *t*-test for statistical analysis (*n* = 3) unless otherwise stated. ****P < 0.0001; ***P < 0.001; ** *P* < 0.01; *P < 0.05; ns, *P* ≥ 0.05.

### TgPDIA3 and TgPDIA6 enhance the re-folding of denatured GFP *in vitro*

Protein folding in the ER, facilitated by molecular chaperones, is critical for achieving correct three-dimensional protein structures. In addition to their redox activity, PDIs can also act as chaperones, assisting in the refolding of proteins that lack disulfide bonds (50).

To evaluate the ability of TgPDIA3 and TgPDIA6 to facilitate protein folding *in vitro,* we utilized a recombinant GFP denatured by acid treatment as a substitute client (51). We cloned, expressed, and purified recombinant soluble protein for TgPDIA3, TgPDIA6, and GFP (Fig. S4A through C). We acid-denatured the recombinant GFP and then exposed it to a renaturing buffer in either the absence or presence of recombinant PDI. We measured protein refolding as a recovery of GFP fluorescence (Fig. 2A). We found that TgPDIA3 significantly accelerated GFP refolding compared to no PDI control (Fig. 2B and C), although the total fluorescence gain remained unchanged (Fig. 2D). TgPDIA6 was less efficient at promoting GFP refolding overall. However, at low concentrations, it produced a significantly greater total fluorescence recovery than the no-PDI control (Fig. 2E through G). For TgPDIA3, the rate of GFP fluorescence recovery, measured as the change in fluorescence over time (ΔF.U./s for the first 7 seconds), was significantly higher at lower TgPDIA3 concentrations than at higher concentrations (Fig. 2H), indicating that TgPDIA3 concentration influences refolding kinetics. TgPDIA6 showed a similar trend, although the recovery rate was lower than that observed for TgPDIA3 (Fig. 2I). Additionally, both TgPDIA3 and TgPDIA6 had a protective effect on the GFP as the fluorescence loss observed after the initial increase was mitigated by the presence of high concentrations of TgPDIA3 (Fig. 2J) and even more so by the presence of high concentrations of TgPDIA6 (Fig. 2K).

**Fig 3:**
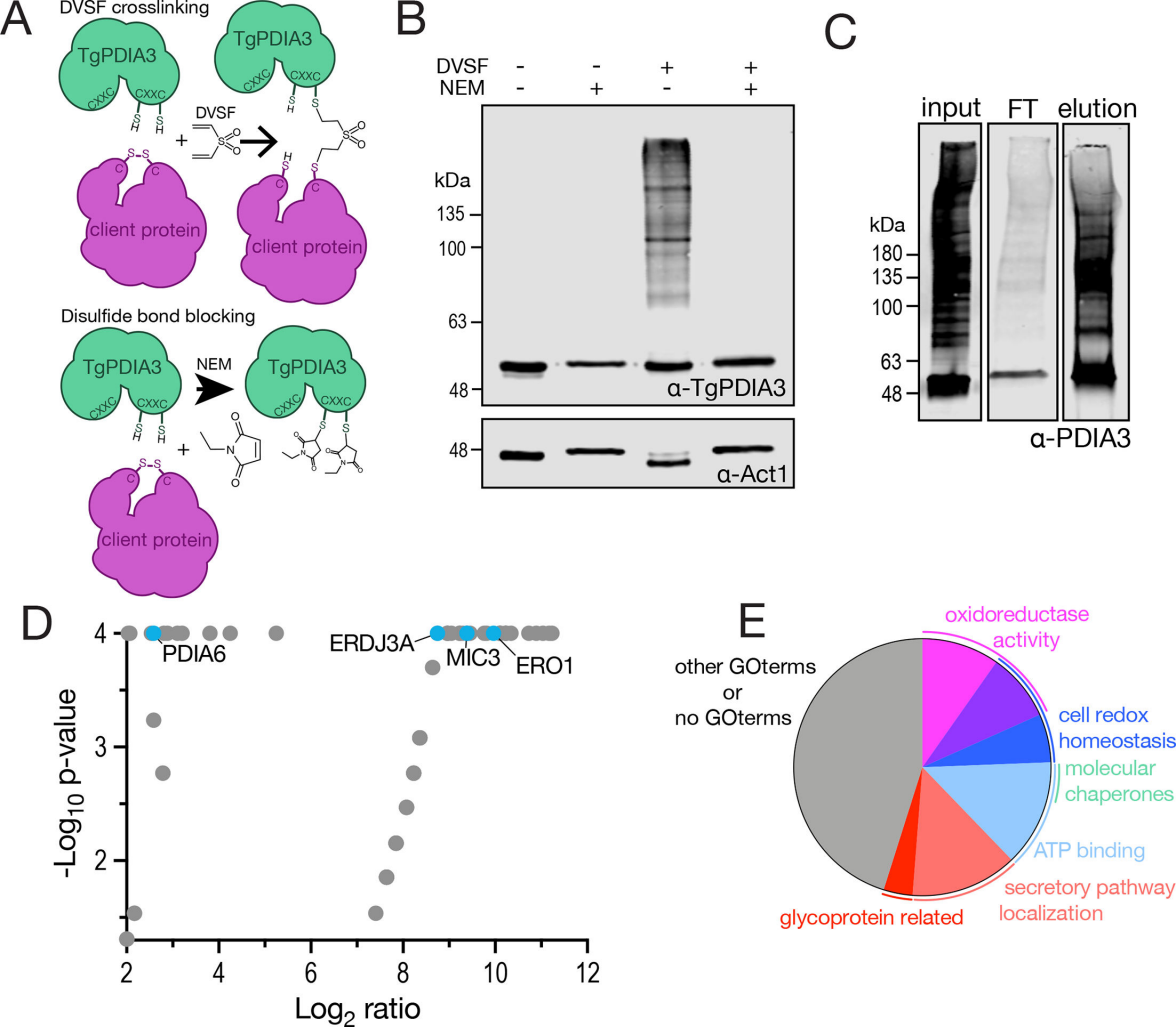
TgPDIA3 is a redox-active PDI with a broad range of substrates. (**A**) Scheme showing how DVSF covalently crosslinks PDIs to their client proteins and how pre-incubation with NEM blocks this interaction. (**B**) Western blots of parasite lysates incubated with DVSF with and without NEM, as indicated in the figure showing *iΔTgPDIA3* bound to potential client proteins (+DVSF/−NEM). Actin1 (α-Act1) was used as loading control. (**C**) Western blots of the pre-IP (input), flowthrough (FT), and +DVSF (elution) from the αTgPDIA3 immunoprecipitation, probed with TgPDIA3 antibody. (**D**) Volcano plot depicting proteins enriched in the +DVSF condition compared to the -DVSF condition with the log_2_ fold change (FC) (*x*-axis) and -Log_10_ *P*-value (*y*-axis). Fisher’s exact test was used for statistical analysis (*n* = 3). (**E**) Pie chart of DVSF-enriched proteins grouped by LOPIT (55) predicted localization, predicted GO terms from ToxoDB, and product description.

To validate that the enhancement of GFP refolding by the PDIs reflects chaperone-like activity rather than redox activity, we performed the refolding assay under reducing conditions. The addition of 1 mM DTT alone improved GFP fluorescence recovery (Fig. S4D and E). Importantly, inclusion of recombinant PDI together with DTT produced a further increase in fluorescence recovery beyond that observed with DTT alone (Fig. S4D and F), demonstrating that the contribution of the PDIs is not dependent on disulfide exchange. To further rule out redox activity, we performed crosslinking with the electrophilic crosslinker divinyl sulfone (DVSF), which covalently links cysteine residues between PDIs and their substrates during disulfide exchange, thereby trapping them together (Fig. 3A) (52–54). We incubated each PDI with denatured GFP in renaturation buffer (± DVSF). Western blot analysis showed no evidence of DVSF crosslinking in the GFP samples, indicating that at the concentrations of PDI used for GFP refolding, no disulfide exchange was detected between the PDI and denatured GFP (Fig. S4G). In summary, TgPDIA3 markedly accelerated GFP refolding, while TgPDIA6 had less effect on the refolding rate but produced a significantly greater total fluorescence gain than the no-PDI control at lower concentrations. These results point to a chaperone role for these PDIs.

### TgPDIA3 is a redox-active PDI with a broad range of substrates

The primary function of PDIs is to catalyze oxidative protein folding by forming, reducing, and rearranging disulfide bonds in client proteins. To investigate the redox role of TgPDIA3, we utilized DVSF (Fig. 3A) (52–54). We incubated *TatiΔku80* parasites with or without DVSF following pre-incubation with or without N-ethylmalemide (NEM), a thiol-reactive compound that blocks disulfide bond formation and serves as a control for DVSF specificity toward cysteine residues (Fig. 3A).

We next analyzed the cell lysates by western blot, probing with the αTgPDIA3 antibody. In the +DVSF/−NEM condition, the observed smear suggested multiple protein interactions, which were absent under +NEM conditions, confirming the specific disulfide exchange of TgPDIA3 with a broad range of substrates (Fig. 3B). To identify these substrates, we incubated *TatiΔku80* parasites with DVSF, immunoprecipitated the lysates using αTgPDIA3-coupled beads (Fig. 3C), and analyzed the captured proteins by liquid chromatography tandem-mass spectrometry (LC-MS/MS). The *TatiΔku80* parental line, incubated without DVSF, was subjected to the same procedure and served as a negative control.

LC-MS/MS analysis revealed 82 proteins significantly enriched in the +DVSF samples compared to the −DVSF control (Fig. 3D). Among these, 20 proteins had gene ontology (GO) terms associated with predicted oxidoreductase activity or cell redox homeostasis (Fig. 3E; Table S4). This group included peroxiredoxins, dehydrogenases, and ER oxidoreductin 1 (ERO1) (TGGT1_300380), an ER redox enzyme that, in mammals, interacts with PDIs (such as HsPDIA3) to oxidize their CXXC motifs and sustain catalytic function (56) (Fig. 3D and Table 1). Among these, several other PDIs were also significantly enriched, including TgPDIA6, which aligns with previous findings that PDIs interact with each other (57, 58). Along with a few other molecular chaperones, the ER resident HSP70 chaperone immunoglobulin heavy chain protein (BiP) was significantly enriched, which aligns with previous literature showing BiP and PDI cooperation in protein folding *in vitro* (59). Interestingly, microneme protein 3 (MIC3) was enriched in the +DVSF condition along with several other proteins predicted to localize within secretory vesicles, suggesting that TgPDIA3 may contribute to the maturation of MIC3 and other secreted effectors through disulfide bond formation (Fig. 3D and Table 1).

**TABLE 1:** Top 20 DVSF-enriched proteins with aTgPDIA3-IP^*a*^

No.	GeneID	Annotated name	Log2FC	LOPIT
1	TGGT1_278830	Glucose-6-phosphate 1-dehydrogenasGe	11.23	Nucleus
2	TGGT1_225850	M28 family peptidase	11.17	ER
3	TGGT1_227948	M16 family peptidase (TLN2)	11.04	Nucleolus
4	TGGT1_215910	Hypothetical protein	10.89	DG
5	TGGT1_228170	IMC2A	10.73	DG
6	TGGT1_277270	NTPase II	10.34	DG
7	TGGT1_300350	Cysteine desulfurase/selenocysteine lyase fam PLP dependent transferase	10.23	ER
8	TGGT1_225050	Putative adenosylhomocysteinase	10.23	Cytosol
9	TGGT1_239752	Hypothetical protein	10.23	Apicoplast
10	TGGT1_311210	Hypothetical protein	10.10	ER
11	TGGT1_271760	Seryl-tRNA synthetase (SerRS2)	10.10	ER
12	TGGT1_272660	Hypothetical protein	10.10	ER
13	TGGT1_300380	Putative ERO1	9.97	ER
14	TGGT1_254490	Sel1 repeat-containing protein	9.81	ER
15	TGGT1_247350	trx domain-containing protein	9.76	ER
16	TGGT1_232250	Catalase	9.45	Cytosol
17	TGGT1_247550	HSP60	9.39	Mito
18	TGGT1_319560	MIC3	9.39	MIC
19	TGGT1_270220	Hypothetical protein	9.39	ER
20	TGGT1_232350	LDH1	9.30	Cytosol

^*a*^ The 20 most enriched proteins identified based on the log2 fold change (FC) in +DVSF compared to −DVSF. The table includes gene IDs, predicted gene products, log2 FC values, and LOPIT-predicted localizations. All *P*-values were <0.0001 (*n = 3*). The complete list of peptides is part of the proteomic results in Table S4.

We next characterized two potential clients of TgPDIA3, TgERDJ3A (TGGT1_209950), a putative thioredoxin predicted to be a PDI, which we chose for its predicted essentiality, and ERO1, which we chose because of its predicted role in refreshing PDI catalytic function, both of which were enriched in the +DVSF condition. We C-terminally HA-tagged TgERDJ3A, inserting its endogenous KDEL retention signal downstream to the tag. Although LOPIT data predicted TgERDJ3A to localize to the apicoplast, it instead localized to the ER as confirmed by co-localization with αTgSERCA (Fig. S5A). Based on CRISPR fitness scores, TgERDJ3A is predicted to be important for parasite fitness (−5.2), while TgERO1 is not (0.82). To assess whether TgERDJ3A and TgERO1 are required for parasite fitness, we inserted a regulatable promoter and an N-terminal 3×Ty-tag into each gene. The respective conditional mutants (*iΔTgERDJ3A* and *iΔTgERO1)* were generated, and their growth phenotypes were analyzed using plaque assays. TgERDJ3A was found to play a significant role in parasite growth, as evidenced by the absence of visible plaques formed by the *iΔERDJ3A* mutant upon addition of ATc to the culture media. In contrast, TgERO1 was non-essential, as the *iΔTgERO1* mutant was unaffected by the presence of ATc (Fig. S5B through D). While TgERO1 was predicted to contain transmembrane domains, TgPDIA6 and TgERDJ3A were predicted to be soluble. We performed membrane extractions to investigate further these predictions (Fig. S5E). We incubated the mutants (*TgPDI6-3HA*, *TgERDJ3A-3HA,* and *TgERO1-3HA*) with DVSF and analyzed the membrane and soluble fractions of each lysate by western blots. The results revealed a unique banding pattern of substrates for each mutant, indicating distinct binding partners and suggesting that different redox-active proteins may have specific client proteins and distinct regulatory roles (Fig. S5E).

To investigate proximal proteins to TgPDIA3 beyond its redox substrates, we C-terminally tagged TgPDIA3 with a modified TurboID construct. To minimize potential mislocalization, the protein’s endogenous GEEL retention signal was inserted downstream to the TurboID sequence (Fig. S6A). We successfully isolated a *TgPDIA3-TID* clonal mutant and validated the tagging by western blot and IFAs using an αHA antibody (Fig. S6B and C).

To confirm the functionality of the TurboID, the *TgPDIA3-TID* mutant was incubated with biotin for 30, 60, and 90 min, followed by western blot analysis probed with streptavidin. We observed a time-dependent increase in biotinylation in the *TgPDIA3-TID* cell line compared with the control (Fig. S6D). The ER-specific localization of biotinylation in the TgPDIA3-TID cells was confirmed through intracellular IFA with Avidin green staining (Fig. S6E).

To increase the specificity of proximal proteins to TgPDIA3, enrich low-abundance ER proteins, and reduce background from highly expressed proteins in the cytosol, plasma membrane (PM), and other organelles, we performed subcellular fractionation followed by gradient centrifugation after biotin incubation (Fig. S6F). The separation of the ER from the PM was validated by western blots of gradient fractions, using PM and inner membrane complex (IMC) markers (αSAG1 and αGAP45, respectively) and ER markers (αTgSERCA and αTgPDIA3) (Fig. S6G). Fractions enriched with ER proteins 6a–7b and the cytosolic S4 fraction were analyzed by LC-MS/MS, along with equivalent fractions of a control cell line obtained from the FBXO14-TID cell line (60), a negative-control strain expressing the cytosolic protein FBXO14 fused to TurboID. The ideal control, an ER-targeted TurboID-only strain to account for non-specific biotinylation in the ER, could not be generated because stable expression of exogenous proteins in the ER could not be reliably achieved. To address this limitation and improve specificity, we isolated ER-enriched fractions using an iodixanol gradient loaded with pre-enriched ER membranes (Fig. S6F). LC-MS/MS analysis revealed 56 proteins enriched in the TgPDIA3-TID ER fraction compared to the FBXO14-TID control (Table S5). Both secretory and ER proteins were enriched in the *TgPDIA3-TID* ER fraction vs. the *FBXO14-TID* cytosolic fraction, including TgSERCA and several microneme and rhoptry proteins (Fig. S6H and I). Interestingly, there were seven proteins that were significantly enriched in both the DVSF-IP and the streptavidin-IP, including MIC3 (Table S6).

In summary, these results demonstrate that TgPDIA3 interacts with multiple substrates in a redox-dependent manner while being proximal to secretory proteins, with MIC3 highlighted as both a proximal partner and a potential substrate.

### TgPDIA3 plays a crucial role in microneme protein secretion and maturation, as well as in rhoptry protein maturation

**Fig 4:**
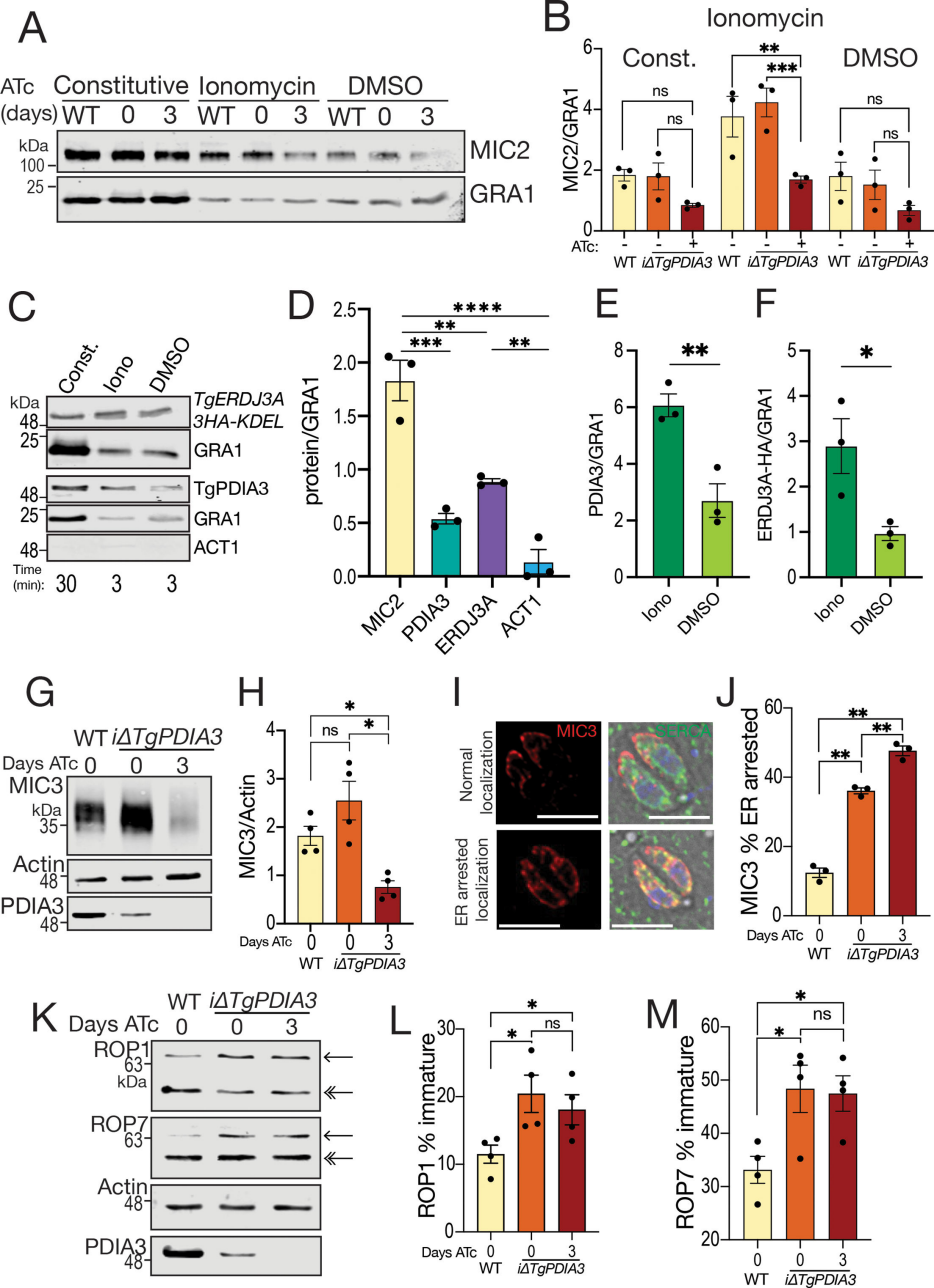
TgPDIA3 impacts microneme protein secretion and maturation, as well as rhoptry protein maturation. (**A**) Representative western blots of microneme secretion assays of *iΔTgPDIA3* ± 2-day ATc pre-incubation, including constitutive (30 min) and induced secretion (3 min) with 1 μM ionomycin or DMSO (control). (**B**) Quantification of standardized band intensity of αMIC2/αGRA1, using ImageStudio. Statistical analysis was performed using two-way ANOVA (*n* = 3). (**C**) Representative western blots of secretions of *TgERDJ3A-3HA-KDEL* or *TatiΔku80,* including constitutive (30 min) and induced secretion (3 min) with 1 μM ionomycin or DMSO (control), probed with αHA and αGRA1, or αTgPDIA3, αGRA1, and αACT1, respectively. (**D**) Quantification of secreted proteins was performed by measuring the band intensity of each antibody signal relative to the αGRA1 signal, including αMIC2, αTgPDIA3, αHA, and αACT1. Statistical analysis was done using one-way ANOVA (*n* = 3). (**E**) Band intensity measurements of induced secretions were quantified by calculating the ratio of the αTgPDIA3 and αGRA1 signals. Statistical significance was assessed using a Student’s *t*-test (*n* = 3). (**F**) Band intensity measurements of induced secretions were quantified by measuring the ratio of the αHA and αGRA1 signals. Statistical significance was assessed using Student’s *t*-test (*n* = 3). (**G**) Representative western blots of *iΔTgPDIA3* ± 3-day ATc pre-incubation and *TatiΔku80* (WT) probed with αMIC3, αACT1, and αTgPDIA3. (**H**) Quantification of the band intensity ratio of αMIC3 to αACT1 in *iΔTgPDIA3* ± 3 days of ATc pre-incubation and *TatiΔku80* (WT). Statistical analysis was conducted using one-way ANOVA (*n* = 4). (**I**) Representative IFAs showing apical MIC3 localization and ER-arrested MIC3 localization. (**J**) Quantification of ER-arrested localization of MIC3 (scale bar, 5 μm). Statistical analysis was conducted using one-way ANOVA (*n* = 3). (**K**) Representative western blots probed with αROP1 and αROP7 in *iΔTgPDIA3* ± 3-day ATc pre-incubation and *TatiΔku80* (WT) showing mature forms (double arrowhead) and immature forms (single arrowhead). Statistical analysis was conducted using one-way ANOVA (*n* = 3). (**L**) Quantification of the percentage of immature protein performed by measuring band intensity of both mature and immature forms (as indicated by the arrows), probed with αROP1. Statistical analysis was conducted using one-way ANOVA (*n* = 4). (**M**) Quantification of the percentage of immature protein was performed by measuring band intensity of both mature and immature forms (as indicated by the arrows) probed with αROP7. Statistical analysis was conducted using one-way ANOVA (*n* = 4). All band intensities were measured using ImageStudioLite from LICOR Bio. ****P < 0.0001; ***P < 0.001; **P < 0.01; *P < 0.05; ns, *P* ≥ 0.05.

*T. gondii* microneme proteins begin their trafficking journey in the ER where they undergo initial processing before progressing through a series of maturation steps, ultimately localizing to the micronemes as fully mature MIC proteins (61). Microneme secretion is triggered by an increase in cytosolic Ca^2+^, and ionophores, such as ionomycin or A23187, stimulate microneme secretion in the absence of host cells (10, 62). Given that TgPDIA3 appears proximal to secretory proteins based on the streptavidin-IP, we next examined the microneme protein secretion and maturation profile of the *iΔTgPDIA3* (±ATc) mutant. We examined first both constitutive secretion of microneme protein 2 (MIC2) over 30 min and secretion induced by ionomycin for 3 min. In the *iΔTgPDIA3* (3 days +ATc) mutant, MIC2 secretion induced by ionomycin was significantly diminished compared to the *iΔTgPDIA3* (−ATc) mutant or the *TatiΔku80* control. Secretion of dense granule protein 1 (GRA1), which is not dependent on calcium, served as secretion control (Fig. 4A and B). This result indicates that TgPDIA3 plays a role in the pathway leading to calcium-induced microneme secretion.

While performing microneme secretion assays, we observed that TgPDIA3 was also secreted. To determine whether its secretion followed similar conditions to those of microneme proteins, we used the same protocol for measuring microneme secretion and analyzed the presence of TgPDIA3 in the culture supernatant by western blot. Supernatants from parental control parasites were probed with αTgPDIA3, and αGRA1 serving as a constitutively secreted control. We also tested another PDI, TgERDJ3A, which was detected by probing a western blot of the *TgERDJ3A-3HA-KDEL* mutant supernatants with αHA. TgERDJ3A was constitutively secreted significantly more than actin, a cytosolic protein used as a control for parasite lysis (Fig. 4C and D). Notably, the secretion of both TgPDIA3 and TgERDJ3A was stimulated by the calcium ionophore ionomycin (Fig. 4C through F).

To further investigate the secretion of TgPDIA3 and TgERDJ3A, we conducted ultrastructure expansion microscopy (U-ExM) experiments to examine the presence of ER structures at the apical end and to determine whether TgPDIA3 partially localizes to the micronemes. We used αMIC2 and αTgPDIA3 to label the micronemes and the ER, respectively. Using Imaris (v9.0) microscopy image analysis software, we generated surface renderings based on fluorescence signals from αMIC2 and αTgPDIA3 staining. Our analysis did not reveal direct colocalization of micronemes (αMIC2) and the ER (αTgPDIA3); however, we observed numerous examples of proximity domains between the two (Video S1). Proximity between the ER and micronemes would ensure that calcium is released precisely where it is required for secretion.

*T. gondii* micronemes proteins are rich in cysteine residues and disulfide bonds (63) (Fig. S7A through C), the formation of which could be catalyzed by PDIs within the ER. To investigate the role of TgPDIA3 in the maturation of microneme proteins, specifically MIC3, which was enriched in both proteomic analyses, we performed western blots with whole cell lysates and probed them with αMIC3 to evaluate the changes in MIC3 protein levels.

We found a significant reduction in total MIC3 in the *iΔTgPDIA3* mutant treated with ATc compared to controls (Fig. 4G and H). To further assess the MIC3 processing defect, we conducted IFAs and observed two distinct patterns of its localization: an apical localization and an ER-arrested form with reduced apical localization. The *iΔTgPDIA3* (+ATc) mutant expressed a higher level of the ER-arrested MIC3 compared to the control (*TatiΔku80*), indicating impaired trafficking of MIC3 (Fig. 4I and J). Further support for the role of TgPDIA3 in MIC3 maturation was provided by its enrichment in the +DVSF condition in the DVSF αTgPDIA3-IP (Table 1). TgPDIA3-mediated disulfide bond formation in MIC3 likely stabilizes it in its correct conformation (Fig. S7B).

Considering the importance of rhoptry proteins for host cell invasion, PV formation, and host immune evasion (9), and their trafficking through the ER, we also explored TgPDIA3’s role in rhoptry maturation. Western blots of parasite lysates probed for rhoptry bulb proteins revealed significantly reduced levels of mature rhoptry bulb protein 1 (ROP1) and rhoptry bulb protein 7 (ROP7) in the *iΔTgPDIA3* mutant, cultured with or without ATc compared to the parental control (*TatiΔku80*) (Fig. 4K through M). Notably, rhoptry maturation was reduced in the *iΔTgPDIA3* (−ATc) mutant, which we attribute to the partial downregulation of *Tgpdia3* due to replacement of its native promoter with a weaker SAG4 promoter. This diminished expression, even in the absence of ATc (Fig. 4G and K), suggests that expression of TgPDIA3 is finely tuned and modest reductions can impact its maturation role of key secretory proteins.

In summary, these findings suggest that TgPDIA3 is essential for the proper folding and maturation of microneme and rhoptry proteins in *T. gondii*. For microneme proteins that are rich in disulfide bonds, this is likely through facilitating disulfide bond formation necessary for structural stability and function. For rhoptry proteins, this could potentially occur through redox modulation of other proteins responsible for secretory protein processing, such as peptidases, which were enriched as redox interactors of TgPDIA3 in the DVSF αTgPDIA3-IP.

### TgPDIA3 regulates TgSERCA and helps maintain low cytosolic calcium levels

The SERCA enzyme is a p-type ATPase that pumps calcium into the ER, helping maintain low cytosolic and high ER calcium levels, both of which are essential for proper cell signaling. Due to this critical role, SERCA activity is tightly regulated by both cytosolic and ER luminal proteins. In vertebrates, PDIs interact with SERCA2b and IP_3_R through redox modifications. When ER calcium levels are high, the activity of SERCA2b is regulated by PDIA3, which forms a disulfide bond with the cysteine residues present in the L4 loop of SERCA2b, inhibiting its activity (30). In vertebrates, this interaction is enhanced by the binding of calreticulin or calnexin to the ER luminal C-terminal tail of SERCA (39). A modified version of the mammalian SERCA2b L4 luminal loop is found in TgSERCA; however, based on homology, the C-terminal calreticulin-interacting tail (64) is absent. Notably, the L4 luminal loop of TgSERCA contains two cysteine residues at positions 947 and 967, mirroring the structure of the human L4 loop in SERCA2b (Fig. S7D through F).

**Fig 5:**
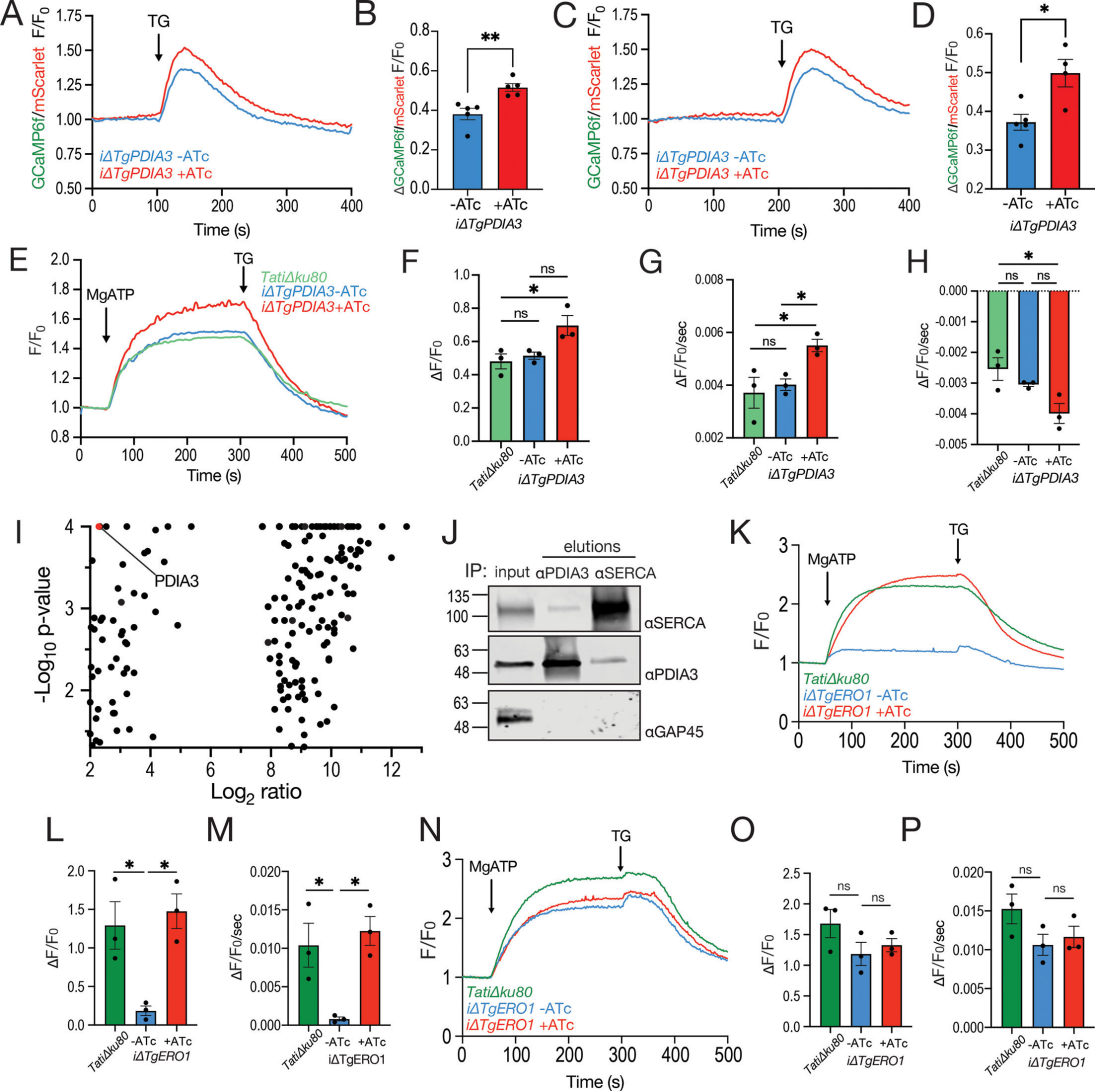
TgPDIA3 regulates TgSERCA. (**A**) Average trace of normalized GCaMP6f/mScarlet fluorescence for cytosolic calcium measurements of the *iΔTgPDIA3* mutant ± 2-day ATc pre-incubation. Thapsigargin (TG; 1 μM) was added at 100 s. (**B**) Quantification of the change in normalized fluorescence after 100 s. Statistical analysis was conducted using Student’s *t*-test (*n* = 3). (**C**) Average trace of normalized cytosolic GCaMP6f/mScarlet fluorescence for calcium measurements of the *iΔTgPDIA3* mutant ± 2-day ATc pre-incubation. Parasites were pre-incubated with 1.8 mM calcium, and 1 μM TG was added at 200 s. (**D**) Quantification of the change in normalized fluorescence after 400 s was performed with statistical analysis conducted using Student’s *t*-test (*n* = 3). (**E**) Average traces of normalized Mag-Fluo-4 fluorescence of the *iΔTgPDIA3* mutant ± 2-day ATc pre-incubation compared to the *TatiΔku80* (control). (**F**) Quantification of the total increase in normalized fluorescence after the addition of 125 μM MgATP. Statistical analysis was done using one-way ANOVA (*n* = 3). (**G**) Quantification of the slope at 50–100 s after the addition of 125 μM MgATP. Statistical analysis was done using one-way ANOVA (*n* = 3). (**H**) Quantification of the slope at 300–400 seconds after the addition of 1 μM TG. Statistical analysis was conducted using one-way ANOVA (*n* = 3). (**I**) Volcano plot showing DVSF-enriched hits for *TgSERCA-3HA* αHA-immunoprecipitation, with the log_2_ ratio fold change (FC) on the *x*-axis and −log_10_ of the *P*-value on the *y*-axis. Fisher’s exact test was used for statistical analysis (*n* = 3). The complete list of enriched peptides is in Table S7. (**J**) Representative western blots of reciprocal immunoprecipitations showing input and 10× concentrated elution from αTgPDIA3- and αTgSERCA-immunoprecipitations, probed with αTgPDIA3, αTgSERCA, and αTgGAP45 (control). (**K**) Normalized fluorescence of Mag-Fluo-4 traces of *iΔTgERO1* mutant ± 2-day ATc pre-incubation compared to *TatiΔku80* (control) with parasites collected extracellularly. (**L**) Quantification of the total increase in normalized fluorescence after the addition of 125 μM MgATP. Statistical analysis was conducted using one-way ANOVA (*n* = 3). (**M**) Quantification of the slope at 50–100 s after the addition of 125 μM MgATP. Statistical analysis was conducted using one-way ANOVA (*n* = 3). (**N**) Normalized fluorescence from Mag-Fluo-4 traces of *iΔTgERO1-OE* mutant ± 2-day ATc pre-incubation compared to *TatiΔku80* (control) with intracellular parasites collected after lysing host cells by passage through a needle. (**O**) Quantification of the total increase in normalized fluorescence after addition of 125 μM MgATP. Statistical analysis was conducted using one-way ANOVA (*n* = 3). (**P**) Quantification of the slope at 50–100 s after the addition of 125 μM MgATP. Statistical analysis was conducted using one-way ANOVA (*n* = 3). ****P < 0.0001; ***P < 0.001; **P < 0.01; *P < 0.05; ns, *P* ≥ 0.05.

We first investigated the impact of TgPDIA3 in ER calcium regulation by assessing cytosolic Ca^2+^ responses to thapsigargin (TG), a SERCA inhibitor, in the *iΔTgPDIA3* (+ATc) mutant. Cytosolic Ca^2+^ changes were measured using the genetically encoded calcium indicator (GECI) GCaMP6f (*K*_*d*_ = 375 nM), which allows assessment of calcium levels through fluorescence changes. We transfected the *iΔTgPDIA3* mutant with a gene expressing GCaMP6f-mScarlet and selected the clone with the highest dynamic range and lowest resting fluorescence (65). Notably, upon addition of thapsigargin, we observed a significantly greater increase in normalized fluorescence in cells cultured with ATc compared to those cultured without ATc, both in the presence or absence of extracellular Ca^2+^. These results indicate that in the absence of TgPDIA3, SERCA activity may be elevated, resulting in increased ER Ca^2+^ content, which in turn resulted in increased Ca^2+^ leakage into the cytosol upon its inhibition with TG. (Fig. 5A through D).

To further explore this phenotype, we examined the potential role of TgPDIA3 in regulating SERCA activity. To this aim, we followed a well-established protocol for ER-calcium uptake mediated by SERCA (66). We loaded the *iΔTgPDIA3* mutant with the low-affinity calcium indicator Mag-Fluo-4 (*K*_*d*_ = 22 µM) for an extended length of time to allow for its compartmentalization (46, 66). Following digitonin permeabilization and washing, parasites were exposed to the SERCA substrate MgATP in the presence of 220 nM free Ca^2+^, and calcium uptake was measured. The resulting fluorescence increase specifically reflected Ca^2+^ uptake by TgSERCA into those organelles where the enzyme is localized, primarily the ER and Golgi (67). Further validation that this activity is due to SERCA Ca^2+^ pumping comes from the effect of thapsigargin, which inhibits SERCA, causing a decrease in Mag-Fluo-4 fluorescence due to calcium leakage from the ER. Upon MgATP addition to a suspension of the *iΔTgPDIA3* mutant (+ATc), we observed a significantly greater change in normalized fluorescence compared to the control strains, *TatiΔku80* and *iΔTgPDIA3* (−ATc) (Fig. 5E and F). This suggests that knockdown of TgPDIA3 resulted in a significant increase in ER calcium uptake by TgSERCA, possibly due to the deficient regulation of TgSERCA at high ER calcium levels. We also observed that the rate of calcium uptake by TgSERCA was significantly higher in the *iΔTgPDIA3* mutant (+ATc) (Fig. 5E and G). After thapsigargin addition, calcium leakage was both faster and greater in the absence of TgPDIA3 (Fig. 5E and H). These findings suggest that TgPDIA3 may regulate TgSERCA activity, which could be through the oxidation of its L4 luminal loop (Fig. S7D through F).

To further substantiate the possible regulation of TgSERCA by TgPDIA3, we performed αHA-immunoprecipitations using a parasite line expressing 3×HA-tagged TgSERCA (*iΔTgSERCA-3HA*), in the presence of DVSF. LC-MS/MS analysis of the samples revealed significant enrichment of TgPDIA3 in the +DVSF condition (Fig. 5I; Table S7). This interaction was confirmed by western blotting using αTgSERCA and αTgPDIA3 in reciprocal immunoprecipitations with αTgPDIA3 and αTgSERCA, respectively (Fig. 5J). Collectively, these data suggest that TgPDIA3 plays a regulatory role in TgSERCA function likely through redox modifications of TgSERCA’s L4 luminal loop.

Considering this finding and the fact that SERCA2b is regulated by multiple redox proteins in vertebrates, we sought to explore whether other ER redox-related proteins might also regulate TgSERCA. We considered TgERO1, which was highly enriched in the LC-MS/MS analysis of the αTgPDIA3 +DVSF sample (Fig. 3D). To investigate the role of TgERO1 in SERCA activity, we used the *iΔTgERO1-3Ty* mutant. Interestingly, we observed an 80% decrease in SERCA activity in the *iΔTgERO1-3Ty* mutant (−ATc). The activity was recovered to the levels of the *TatiΔku80* control when the *iΔTgERO1-3Ty* mutant was pre-incubated with ATc (Fig. 5K through M). Previous studies have shown that ERO1 overexpression, especially in the absence of SEPN1, can lead to excessive production of H_2_O_2_ in the ER. This causes hyperoxidation of the L4 luminal loop of TgSERCA, severely decreasing its activity (68). RNA-seq data from toxodb.org show that *Tgero1* (*TGME49_300380*) is expressed at 52 TPM (transcripts per million) in tachyzoites, whereas *sag4* (*TGME49_280570*), from which the promoter domain was used for the generation of the *iΔTgERO1-3Ty* line, is expressed at 1692 TPM (69). This represents a 32-fold higher transcription level compared to endogenous *Tgero1*, suggesting that TgERO1 could be overexpressed in the *iΔTgERO1-3Ty* mutant (-ATc), explaining the observed loss of SERCA activity. TgERO1 was not enriched in the +DVSF condition of the *iΔTgSERCA-3HA* αHA-immunoprecipitation (Table S7), indicating that the reduction of SERCA activity was not through direct disulfide exchange with ERO1. Because TgSERCA is essential for parasite viability, we questioned whether the minimal activity observed in the *iΔTgERO1-3Ty* (−ATc) mutant reflected stress associated with parasite preparation and loading in extracellular conditions, especially since the mutant displayed no obvious growth defect. To test this, we measured SERCA activity in intracellular parasites by releasing them by needle passage and maintaining them in intracellular buffers. Under these conditions, SERCA activity did not differ between the *iΔTgERO1-3Ty* (−ATc) mutant and the controls (Fig. 5N through P).

The ER is one of the main sites of ATP consumption, and in mammals, some redox proteins are active at mitochondria-associated membranes (MAMs) to regulate Ca²⁺ transfer from the ER to the mitochondria in order to increase ATP production or induce apoptosis during prolonged ER stress (28). Since there is a defect in ER calcium regulation in the *iΔTgPDIA3* mutant (+ATc), we next explored the potential role of TgPDIA3 in calcium transfer from the ER to the mitochondrion. To test this, we transfected the *iΔTgPDIA3* mutant with a GCaMP6f-SOD2 plasmid, which allows the expression of GCaMP6 in the mitochondrion (65, 70, 71) (Fig. S8A). Since the *T. gondii* mitochondrion does not respond to cytosolic Ca²⁺ increases resulting from extracellular Ca²⁺ entry (46) and instead relies on Ca²⁺ accumulation in ER-mitochondrion microdomains (46), we used thapsigargin, which inhibits SERCA, allowing the accumulation of Ca^2+^ on the cytosolic side of the ER membrane. Calcium transfer into the mitochondrion upon the addition of thapsigargin was observed as an increase in the GCaMP6 fluorescence, which was lower for the *iΔTgPDIA3* mutant, although not statistically significant. This result suggested a potential defect in the transfer of calcium from the ER to the mitochondrion (Fig. S8B and C). We also used zaprinast, a phosphodiesterase inhibitor, which causes the release of calcium from the ER and acidic organelles. In the *iΔTgPDIA3* mutant (+ATc), there was significantly less calcium taken into the mitochondria compared to the control (−ATc) (Fig. S8D and E). A similar trend was observed following the addition of glycyl-L-phenylalanine 2-naphthylamide (GPN) (Fig. S8F and G), a lysomotrophic agent that mobilizes calcium from acidic stores (72) like the plant-like vacuolar compartment or PLVAC (73, 74), an acidic compartment with lysosomal and secretory functions. This result was unexpected because SERCA is not properly regulated in the *iΔTgPDIA3* mutant, leading to increased Ca^2+^ uptake into the ER. We therefore anticipated either no change or an increase in Ca^2+^ transfer to the mitochondrion. One possible explanation is that the ER leaks calcium in a more diffused manner rather than specifically at ER-mitochondria contact sites, resulting in greater calcium dilution in the cytosol. This hypothesis is supported by the cytosolic calcium response shown in Fig. 5A and B. Despite this, there was no change to mitochondrial membrane potential in the *iΔTgPDIA3* mutant compared to controls (Fig. S8H).

In summary, our results indicate that TgPDIA3 regulates TgSERCA activity, which may, in turn, influence calcium levels in the cytosol, ER, and mitochondria.

## DISCUSSION

**Fig 6:**
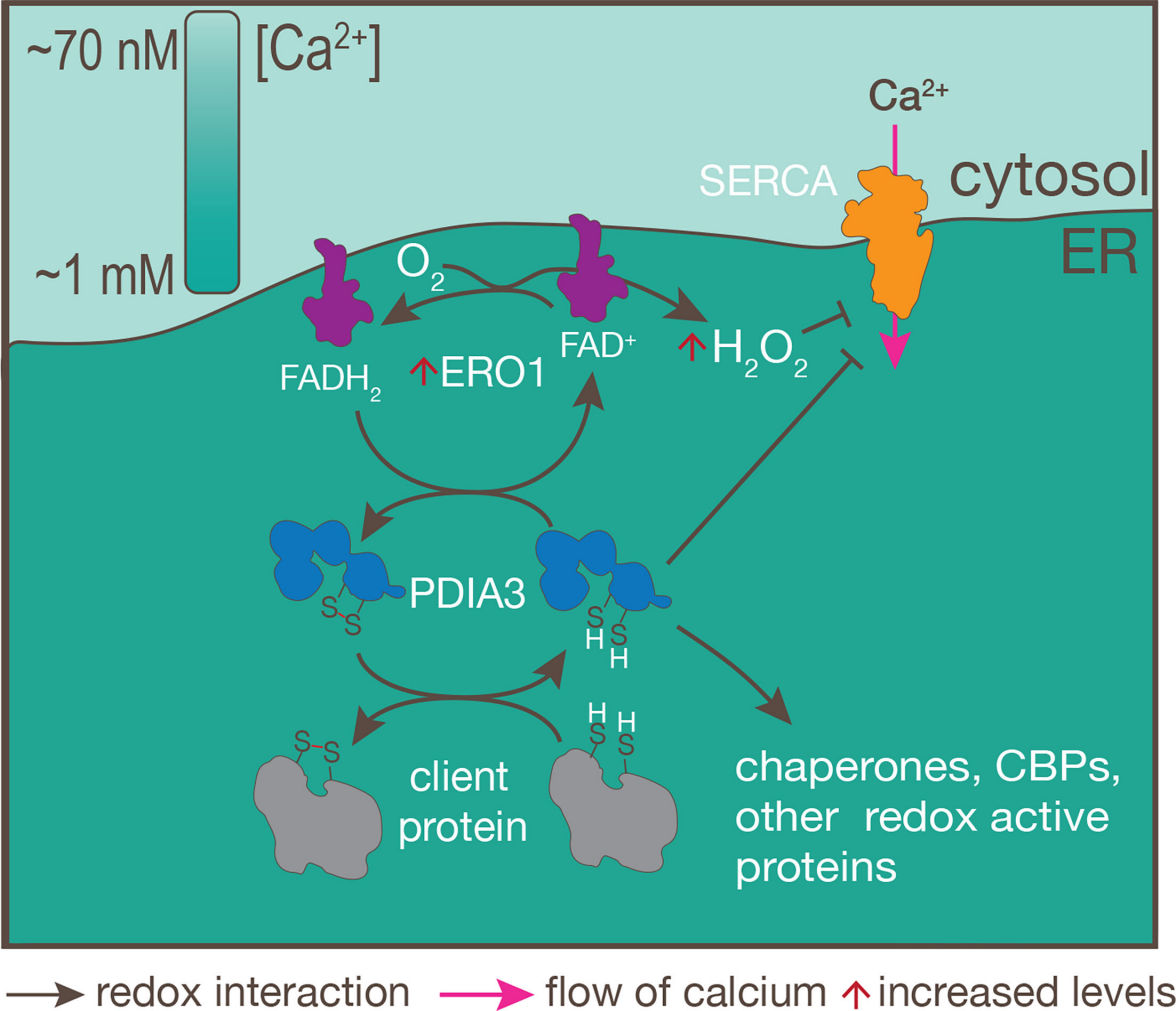
Model of the role of TgPDIA3 in the ER. TgPDIA3 interacts with client proteins and catalyzes disulfide bond formation. Oxidized TgPDIA3 is regenerated by TgERO1, a process that produces hydrogen peroxide as a by-product. The model also shows redox regulation of TgSERCA by TgPDIA3 under conditions of elevated ER calcium and by reactive oxygen species generated upon TgERO1 overexpression. Additional potential TgPDIA3 interactions are suggested based on proteins enriched in the DVSF-IP proteomic analysis.

In this study, we characterized several redox proteins of the *T. gondii* ER, including TgPDIA3 (*TGGT1_211680*), TgPDIA6 (*TGGT1_249270*), TgERO1 (*TGGT1_300380*), and TgERDJ3A (*TGGT1_209950*). We demonstrated the protein folding ability of TgPDIA3 and TgPDIA6 and established the role of TgPDIA3 in microneme secretion and in the maturation of microneme and rhoptry proteins. TgPDIA3 was shown to be a catalytically active protein disulfide isomerase with a distinct set of substrates. Both TgPDIA3 and TgERO1 modulated the calcium-pumping activity of TgSERCA, which was further supported by the demonstration of TgPDIA3-SERCA interaction. Finally, the redox activity of TgPDIA3 impacted ER calcium homeostasis, which, in turn, affected calcium transfer to the mitochondrion (Fig. 6).

Denatured GFP served as an effective substrate for testing PDI refolding activity, with fluorescence recovery providing a quantitative measure of refolding efficiency. Using this assay, we demonstrated that both recombinant TgPDIA3 and TgPDIA6 promoted the refolding of denatured GFP, restoring its fluorescence. Although GFP contains two cysteine residues, it does not form disulfide bonds in its native, properly folded state (75). This makes it unlikely that the observed enhancement of GFP refolding is mediated by the formation of native disulfide bonds, a conclusion that we demonstrated by including a control using the reducing agent DTT. Denaturation may expose cysteine residues that can engage in non-native intra- or intermolecular disulfide bonds during refolding, trapping GFP in misfolded or aggregated states. The enhancement of GFP refolding by DTT is consistent with the maintenance of cysteines in a reduced state that permits productive folding. Importantly, the additional improvement observed in the presence of PDIs even under reducing conditions indicates that the contribution of TgPDIA3 and TgPDIA6 reflects chaperone-like activity rather than redox-dependent catalysis. This effect may result from interactions between unfolded substrate proteins and hydrophobic residues located within the *b* and *b′* domains of the PDIs, which have been shown *in vitro* to reduce aggregation and promote folding (50). Further experiments are needed to confirm this possibility.

Previous studies have used DVSF to capture redox interactions between PDIs and their substrates in apicomplexans (76). Using a DVSF αTgPDIA3-IP approach, we identified numerous potential redox partners of TgPDIA3, including other PDIs, suggesting that *T. gondii* PDIs may interact with each other similarly to their mammalian counterparts (57, 58). The enrichment of TgERO1 in the DVSF αTgPDIA3-IP provides a potential mechanism for PDI cysteine re-oxidation, similar to mammalian PDIs, where ERO1 likely oxidizes disulfide bonds on PDI to sustain its catalytic function (56, 77).

Interestingly, metallopeptidases were among the most significantly enriched proteins in the DVSF αTgPDIA3-IP, which represents a previously unreported interaction that may indicate a regulatory role for TgPDIA3 in peptidase activity. However, further studies are needed to determine the functional significance of this interaction. Metallopeptidases have been previously studied in *T. gondii* (78–81), and several characterized members have been shown to be secreted, and are thought to cleave host proteins. We also observed enrichment of Cdc48, suggesting a possible involvement of TgPDIA3 in ER-associated degradation (ERAD) (82). Additionally, several proteins predicted as glycosyltransferases were identified, aligning with the known function of mammalian PDIA3 in catalyzing disulfide bond formation in glycoproteins. Future work on these TgPDIA3 substrates is warranted to better understand their functional significance in *T. gondii* biology. Understanding the molecular mechanisms of these interactions could reveal new insights into redox regulation, protein folding, and ER-associated processes in the parasite.

Apicomplexans are named for their apical complex, a critical assembly of structures and organelles that secrete effectors essential for the various phases of the parasite’s lytic cycle (6, 7). Micronemes and rhoptries, specialized secretory organelles located within the apical complex, play crucial roles in the invasion of host cells (83). *T. gondii* secreted proteins from micronemes and rhoptries go through multiple modifications in various subcellular compartments before reaching their mature forms (84). *TgPDIA3-TID* LC-MS/MS results showed an enrichment of secreted proteins, suggesting that TgPDIA3 interacts closely with *T. gondii*-secreted effectors, likely as they are trafficked through the ER (61). In this regard, a recent study about the characterization of TgPDIA3 (aka TgPDI8) also found that it was proximal to secretory proteins (85). Our study found that TgPDIA3 is important for the maturation of selected microneme and rhoptry proteins, including MIC3, ROP1, and ROP7. We hypothesize that TgPDIA3 facilitates this maturation through disulfide bond formation, especially for MIC3, which was significantly enriched in the DVSF αTgPDIA3-IP. For rhoptry proteins, which contain fewer disulfide bonds and were not enriched as redox substrates of TgPDIA3, the role in maturation could be indirect, potentially through redox modulation of other proteins, such as peptidases, several of which were enriched as TgPDIA3 redox interactors in the DVSF αTgPDIA3-IP. Since parasite invasion, gliding, and egress all depend on microneme secretion, and this secretion is tightly regulated by intracellular calcium signaling (10, 62), TgPDIA3 may also contribute to these processes indirectly. As the largest intracellular calcium store, the ER is responsible for calcium distribution and forms connections with other calcium stores like the mitochondrion and the PLVAC (46). The ER of *T. gondii* forms a vast, interconnected tubular network that extends throughout the entire parasite (86). U-ExM experiments revealed close proximity between the ER and micronemes (Video S1), suggesting a potential role for calcium release from the ER in domains adjacent to micronemes, facilitating calcium-induced microneme secretion. The presence of calcium microdomains between the ER and other organelles is well established (87, 88) and serves to prevent global cytosolic calcium increases that could lead to cytotoxicity or unintended signaling. Because microneme proteins mature while trafficking through vesicular compartments from the ER to the Golgi (61), their transport and maturation are unlikely to depend on ER-microneme proximity. However, ER-microneme proximity could play a role in the localized release of calcium from the ER, which may be important for microneme secretion. This spatial arrangement may provide an advantage to the parasite by enabling rapid and localized calcium release to micronemes without the need for global cytosolic calcium elevation. In this regard, we found that TgPDIA3 downregulation resulted in reduced calcium-induced microneme secretion while constitutive secretion showed no significant difference. The reduced regulation of SERCA in the *iΔTgPDIA3* mutant further supports our hypothesis, as increased TgSERCA activity would be expected to lower the calcium concentration in the ER-microneme microdomain. This effect could account for the invasion defect observed in the *iΔTgPDIA3* mutant.

In mammals, some ER-resident proteins, such as BiP and calreticulin, have been shown to be secreted during calcium-induced ER stress (89). Calreticulin has been proposed as an “eat-me” signal in dead or dying cells and can also promote the phagocytic uptake of cells undergoing ER calcium depletion (90). Additionally, impaired PDI secretion from injured endothelial cells has been linked to defective blood clot formation (91, 92). HsPDIA3, specifically, has been associated with extracellular matrix mineralization through its interaction with bioactive vitamin D and bone morphogenetic protein-2 (BMP2) (93, 94). In this context, we found that both TgPDIA3 and TgERDJ3A were secreted in response to elevated calcium. Previous studies have proposed TgPDIA3 as a diagnostic marker for *T. gondii* infection based on its recognition by IgA antibodies in human tears and milk (95). Based on its abundance, secretion potential, and preliminary data, TgPDIA3 may also represent a promising candidate for *T. gondii* vaccine development (96).

Previous studies have demonstrated that calcium movements in the vertebrate ER are redox-regulated through cysteine modifications of various ER membrane proteins involved in calcium transport (29, 30). Our lab previously showed that TgSERCA is essential for replenishing ER calcium during the lytic cycle and facilitates its transfer to other compartments, ensuring the replenishment of all intracellular calcium stores (46).

The *iΔTgPDIA3* mutant showed increased TgSERCA-mediated calcium uptake and accelerated Ca²⁺ leakage following SERCA inhibition with thapsigargin, supporting a regulatory role of TgPDIA3 in TgSERCA activity (30). We hypothesize that this regulation occurs via direct redox modification of TgSERCA’s L4 luminal loop between cysteine 947 and cysteine 967 by TgPDIA3 under conditions of high ER calcium. Supporting this hypothesis, TgPDIA3 was enriched in the DVSF *TgSERCA-HA* αHA-IP. Interestingly, overexpression of TgERO1 caused a marked reduction in SERCA activity in extracellular parasites, likely due to hyperoxidation of TgSERCA’s luminal L4 loop resulting from excessive H_2_O_2_ production (68). In the *iΔTgERO1* (−ATc) mutant, TgERO1 could be overexpressed as a result of the replacement of its endogenous promoter with a stronger one. Interestingly, the *iΔTgERO1* (−ATc) mutant displayed normal growth despite the critical role of TgSERCA (46). However, when the same SERCA activity experiment was performed with intracellular parasites (obtained by needle-lysing parasite-filled cultures), no significant difference in SERCA activity was observed between the *iΔTgERO1* (−ATc) parasites and controls. Because H_2_O_2_ freely diffuses across membranes and the mitochondrion of intracellular parasites expands the length of the parasite, likely forming multiple contacts with the ER, while the mitochondrion of extracellular parasites is collapsed (97), we hypothesize that H_2_O_2_ overproduction by ERO1 overexpression may be mitigated by mitochondrial antioxidants, such as peroxiredoxins, in intracellular parasites. Alternatively, while SERCA may function normally during intracellular replication, the stress of parasite purification and exposure to extracellular conditions could trigger elevated H_2_O_2_ production by overexpressed ERO1, resulting in a sharp inhibition of SERCA activity. Future experiments will focus on measuring H_2_O_2_ levels in extracellular *iΔTgERO1* (−ATc) parasites, comparing conditions with and without ATc to assess the impact of ERO1 overexpression.

It is interesting that despite increased calcium leakage into the cytosol, mitochondrial calcium uptake was diminished rather than elevated. PDIs are known to localize to mitochondria-associated membranes (MAMs) (98) and may facilitate Ca²^+^ transfer from the ER to the mitochondrion. Our experiments showed reduced mitochondrial Ca²^+^ uptake in the *iΔTgPDIA3* (+ATc) mutant, which we hypothesize is due to a diffused Ca²^+^ leakage from the ER after inhibition of SERCA rather than being localized to specific microdomains between the ER and mitochondrion. Importantly, the mitochondrial membrane potential was not affected in the mutant, indicating that the reduced Ca²^+^ uptake is not due to impaired mitochondrial function. It is possible that TgPDIA3 may be important for the localization of specific proteins involved in the transfer of Ca²^+^ between the ER and the mitochondria. More work is needed to characterize this phenomenon.

In conclusion, our study highlights the critical role of TgPDIA3 in the redox regulation of calcium signaling in *T. gondii*. By modulating ER calcium homeostasis, TgPDIA3 influences key processes such as microneme secretion, which is essential for parasite invasion. We also identified several TgPDIA3 substrates and demonstrated their involvement in parasite replication and the maturation of secreted proteins. These findings represent the first evidence of the link between redox regulation and calcium homeostasis in *T. gondii*, expanding our understanding of the role of the *T. gondii* ER in Ca²^+^ storage and signaling. Calcium signaling is crucial for the progression of the lytic cycle (99–101), with intracellular stores playing a key role in this process (46, 102). Future studies will help elucidate the mechanisms by which the ER regulates Ca²^+^ storage and release.

## MATERIALS AND METHODS

### Phylogenetic analysis

Sequences were obtained through the NCBI database and VEupath DB (103). Sequences were aligned in MEGA-X using Clustal W with manual trimming: pre-trimming length of 830 and post-trimming length of 623. Maximum likelihood trees were also constructed on MEGA-X (104). A phylogeny test was conducted using the bootstrap method, with 1,000 bootstrap replications. Amino acid substitution was modeled using the Jones-Taylor-Thornton (JTT) model. Uniform rates and all sites were used in tree construction. The nearest-neighbor-interchange (NNI) maximum likelihood heuristic model was used.

### Cell culture

Human telomerase reverse transcriptase (hTERT) fibroblasts (105) were cultured in Dulbecco’s modified Eagle medium (DMEM-HG) with 10% bovine calf serum (BCS) at 37°C with 5% CO_2_. Human foreskin fibroblasts (HFF) cells were cultured in DMEM-HG with 15% FBS at 37°C with 5% CO_2_. *T. gondii* tachyzoites were cultured in hTERT fibroblasts in DMEM-HG with 1% BCS at 37°C with 5% CO_2_. C-terminally tagged mutants were grown in media supplemented with 6.8 µg/mL chloramphenicol, and promoter insertion mutants were grown with 1 µM pyrimethamine. For the downregulation of target genes, parasites were grown with 0.5 µg/mL anhydrous tetracycline.

### Generation of plasmids and mutants

For the generation of Cas9 plasmids, primers (primer sequences are listed in Table S3) containing guide RNAs (gRNAs) corresponding with the untranslated region upstream (for promoter insertion) or downstream (for C-terminal tagging) of a gene of interest were used to amplify the Cas9 plasmid using Q5 PCR, followed by KLD treatment and bacterial transformation as previously described (106). Plasmids with modified retention signals were also altered using Q5 PCR and site-directed mutagenesis. Recombinant protein plasmids were created using Gibson assembly (107). All inserted sequences were confirmed using Sanger sequencing.

Gene tagging was performed using a CRISPR/Cas9 approach. For each construct, *TatiΔku80* parasites were transfected with a Cas9 plasmid encoding a guide RNA (gRNA) specific to either the 5′ or 3′ untranslated region (UTR) immediately flanking the gene of interest (GOI). Corresponding repair templates were amplified with homology regions located just inside the gene of interest (GOI) and on the opposite side of the gRNA target site to introduce either a regulatable promoter and/or an N-terminal tag at the 5′ end or a C-terminal tag at the 3′ end of the GOI. Promoter insertion and tagged mutants were enriched with drug selection, subcloned, and validated genomically via PCR, and protein tagging was validated via IFA and western blots. Fluorescent mutants, which were created by transfection of tdTomato, GCaMP6f-mScarlet, or linearized GCaMP6f-SOD2 (65), were enriched through fluorescence sorting on a BioRad S3 cell sorter, and the fluorescence of parasites in the mixed populations was validated via live cell imaging on a DeltaVision, DVElite. All mutants were subcloned to obtain single clones after multiple rounds of enrichment. To select GCaMP6f clones, the fluorescence of 2 × 10^7^ parasites was measured on a BioTek Synergy H1 Hybrid Reader, and 1 µM ionomycin was added to determine calcium dynamic range. Clones with the lowest resting fluorescence and the largest dynamic range were selected and used for the experiments.

### Gene cloning and protein purification

*Tgpdia3* (*TGGT1_211680*) and *tgpdia6* (*TGGT1_249270*) were amplified from *T. gondii* RH cDNA, and GFP was amplified from the pTREX-GFP plasmid. The fragments were cloned into the pQE-80L vector by Gibson Assembly, a 5’ exonuclease-3’ extension DNA assembly method, and constructs were transformed into NEB 5α competent *E. coli.* For each gene, two of these bacterial colonies were sent for sequencing for confirmation of proper assembly. The plasmids were then transformed into protein-expressing bacteria, either BL21 or BL21 codon+ *E. coli,* and colonies were tested for IPTG-induced gene expression. Protein expression and solubility were optimized by altering IPTG concentrations, induction temperatures, and times. Protein induction and solubility were visualized on a Coomassie-stained gel (Fig. S3B, S4A and B), and western blots of the induced and uninduced samples were probed with αHis antibody at 1:1,000 to determine solubility of the proteins. Soluble proteins were purified using a HisPur Ni-NTA Chromatography Cartridge (CAT:90098) as previously described (49).

### Generation of antibodies in mice

The antigen mixture was prepared using the purified recombinant protein described above and either Freund’s Complete adjuvant (CAT: F5881) (for initial injection) or Freund’s incomplete adjuvant (CAT: F5506) (for subsequent booster injections). Mice were intraperitoneally injected with the antigen once every 2 weeks for a total of four injections; 100 µg of protein per mouse was used for the primary inoculation, and 50 µg of protein per mouse was used for the boosts. Serums were collected after each injection to test for the presence of antibodies. Mice were euthanized before blood collection which was stored at 4°C overnight to allow for separation of serum (49, 108).

### Immunofluorescence assays

Immunofluorescence assays were conducted as previously described (73). Tachyzoites were grown on confluent HFF or hTERT cells on coverslips for ~24 h. Cells were either fixed and permeabilized with 100% methanol for 2 min, followed by rehydration with PBS or fixed with 3% paraformaldehyde and permeabilized with 0.25% Triton X-100 in PBS. Coverslips were blocked and incubated with antibodies in 3% bovine serum albumin (BSA) in PBS pH 8.0. Coverslips were washed with PBS pH 8.0 after blocking, primary antibody incubation (mouse αTy, 1:200 from Drew Ethridge; rat αHA at 1:50, Roche) and secondary antibody incubation. Cells were imaged using a DeltaVision, DVElite.

### Western blots

For general protein lysis, parasites were incubated with Cellytic M (CAT: C2978) (with DNase and RNase) for 5 min at RT. Proteins were then boiled with Laemmli BME for 5 min. For western blots, SDS-PAGE-separated proteins were transferred to a nitrocellulose membrane. Membranes were blocked with 5% milk in PBS-T before incubating with primary antibodies in PBS-T, followed by secondary antibodies in PBS-T. Blots were washed with PBS-T between steps and before imaging and were imaged using a LiCOR Odyssey CLx imager.

### Plaque assays

Plaque assays were performed as previously described (109, 110). Freshly egressed tachyzoites were collected, filtered, and counted; 200 tachyzoites/well were used to infect confluent hTERT monolayers in 6-well plates and incubated at 37°C for 7 days. Mutant and control strains were incubated with or without ATc, after which monolayers were fixed for 5 min with 100% ethanol, stained with crystal violet for 5 min, and washed with PBS. Plates were imaged and plaque sizes were measured using FIJI (111); 16 plaques were measured per biological replicate and three biological replicates were used per condition.

### Growth and replication assays

Growth assays were performed as previously described (112). Confluent monolayers of hTERT cells in a 96-well plate were infected with freshly egressed tdTomato-expressing tachyzoites at 4,000 parasites per well. Mutants were incubated either with or without ATc, and fluorescence was measured daily on a BioTek Synergy H1 Hybrid Reader for 8 days to track parasite growth. A standard curve was created using serial dilutions of fluorescent parasites in order to calculate the number of parasites per well based on their fluorescence level.

Replication assays were conducted as previously described (108), with a few modifications; 5 × 10^5^ freshly egressed RFP-expressing tachyzoites were used to infect confluent hTERT fibroblast monolayers on coverslips. Coverslips were fixed and mounted 20 hpi. The number of 1, 2, 4, and 8+ parasite-containing vacuoles was counted until >100 total vacuoles were counted per condition and quantified.

### Invasion (green/red) assay

Invasion assays were conducted as previously described (48), with a few modifications (49); 2 × 10^7^ of freshly egressed RFP-expressing tachyzoites were washed and resuspended in invasion media (3% FBS, 10 mM HEPES, in DMEM-HG, pH 7.4) and allowed to settle on HFF-coated coverslips for 20 min on ice. Plates of parasites were placed in a 37°C bath for 5 min to stimulate invasion and then returned to ice and immediately fixed with 3% paraformaldehyde to stop invasion. Coverslips were blocked and probed with rabbit αSAG1 at 1:1,000 (a gift from Vern Carruthers) to differentiate the extracellular parasites from the intracellular ones. Parasites were then counted, and the number of red parasites (all) and green (extracellular) was obtained and graphed as a percentage of total parasites that invaded.

### *In vitro* protein folding assays

To evaluate the protein chaperoning abilities of TgPDIA3 and TgPDIA6, we adapted a protein chaperone assay utilizing recombinant TgPDIs and a recombinant acid-denatured GFP (51). All proteins were diluted and concentrated in PBS to remove contaminating imidazole from the purification process (because it has chaperoning abilities on its own). Recombinant GFP was diluted in denaturing buffer (0.3 mM EDTA, 50 mM Tris-HCl, pH 7.5) and mixed with an equal volume of 125 mM HCl to acid-denature GFP for 1 min at RT. Denatured GFP was added to a cuvette containing renaturation buffer (25 mM MgCl_2_, 100 mM KCl, 50 mM Tris-HCl, pH 7.5) either with or without PDI after 20 s of initial reading. Re-folding was measured as a gain in fluorescence using a Hitachi F-7000 Fluorescence Spectrophotometer (51). Spontaneous refolding in the absence of PDI was compared to refolding in the presence of different concentration ratios of GFP to PDI; 1 mM dithiothreitol (DTT) was used as a disulfide reducing control.

### DVSF crosslinking

DVSF crosslinking was conducted as previously described (76), with a few modifications. In brief, the chemical crosslinker divinyl sulfone (DVSF), 97% (Thermo Cat Number: L12827-09), was used to covalently bind PDI’s catalytically active cysteines in the CXXC motif with cysteine residues in client proteins during disulfide bond exchange. For blocking disulfide bond formation and to show DVSF specificity, the indicated samples were pre-incubated with 1 mM N-ethylmaleimide (NEM) for 3 h in culture prior to parasite collection. DVSF was diluted to a concentration of 3 µL DVSF/10 mL buffer A with glucose (BAG) (116 mM NaCl, 5.4 mM KCl, 0.8 mM MgSO_4_, 5.5 mM d-glucose, and 50 mM HEPES, pH 7.4), and freshly collected parasites were resuspended in 1 mL of BAG with DVSF or BAG without DVSF and incubated in a 37°C water bath with rotation for 30 min. Parasites were then centrifuged and washed once with BAG before centrifugation and resuspension in lysis buffer. Parasites were lysed, and proteins were collected for western blot analysis. For the enrichment of membrane proteins, cells were subjected to three freeze-thaw cycles, and lysates were centrifuged to separate soluble (supernatant) and membrane (pellet) proteins. Proteins were then solubilized using 1% SDS or 1% NP-40 for subsequent analysis.

### TgPDIA3 co-immunoprecipitation

The IP was carried out as previously described with some modifications (113). For antibody-bead coupling, protein G magnetic beads (Thermo Cat. number: 88847) were gently vortexed to resuspend, and 100 µL was washed, collected, and incubated with mouse αTgPDIA3 antibody in 1× coupling buffer. The 1× coupling buffer was prepared from a 20× stock (200 mM NaH₂PO₄, 3 M NaCl, pH 7.2) supplemented with 5% fresh lysis buffer (150 mM NaCl, 20 mM Tris, pH 7.6, 0.1% SDS, 1% Triton X-100). The antibodies were crosslinked to the beads using 220 mM DMP (Sigma Cat Number: D8388) in 200 mM sodium borate, pH 9.0. The coupling was quenched using 200 mM ethanolamine, pH 8.5.

Parasites were collected as described above for DVSF crosslinking and lysed for 5 min on ice in a lysis buffer containing protease inhibitors (Thermo Cat Number: 11836170001) with periodic vortexing. Lysates were centrifuged at 21,000 × *g* for 5 min at 4°C, and the supernatant was added to the αTgPDIA3-coupled beads and incubated at 4°C overnight. The unbound protein was then collected, and the beads were washed with lysis buffer three times before boiling with 1× Laemmli for western blot analysis or were frozen dry and sent for LC-MS/MS analysis.

### *TgSERCA-3HA* co-immunoprecipitation

Parasites were collected as previously described for DVSF crosslinking, including the enrichment of membrane proteins step. After freeze-thaw, the initial pellets were resuspended, followed by the removal of soluble proteins. Pellets containing the membrane proteins were lysed for 5 min on ice in lysis buffer (50 mM HEPES, pH 7.4, 50 mM NaCl, 1% NP-40, and mini complete protease inhibitor [Cat Number: 11836170001]), with periodic vortexing. Lysates were centrifuged at 21,000 × *g* for 5 min at 4°C. After washing 30 µL of Pierce α-HA magnetic beads (Cat Number: 88836) three times with lysis buffer, the clarified lysates from 4 × 10⁸ parasites were added to the beads and incubated overnight at 4°C. The beads were collected, and unbound proteins were removed. The beads were then washed with lysis buffer three times before boiling with 1× Laemmli (in lysis buffer) for western blot analysis, or were frozen dry and sent for LC-MS/MS analysis.

### Subcellular fractionation and gradient centrifugation

Subcellular fractionation was carried out as previously described (73, 114). Following a 1 h incubation with 50 μM biotin at 37**°**C, parasites were collected, needle lysed out of host cells, and filtered through an 8-µm nuclepore membrane (73). Parasites were then washed with BAG, counted, and lysed by grinding with silicone carbide on ice for 30-s intervals with 30-s pauses in between for a total grinding time of approximately 2 min, in order to keep organelles intact. Parasites and silicon carbide were resuspended in lysis buffer (50 mM KCl, 4 mM MgCl_2_, 0.5 mM EDTA, 20 mM HEPES-KOH [pH 7.2], 125 mM sucrose, mini-complete protease inhibitor, 12 µg/mL DNase, 12 µg/mL RNase, and 8 µg/mL nocodazole). Silicon carbide was removed through a series of low-g centrifugations. Fractions were collected after each centrifugation step (Fig. S6F). Pellets and supernatants were collected, and supernatants were re-spun for the following centrifugation until the pellet (P3) fraction was obtained, which was homogenized and mixed with the 20% layer of an Optiprep (Abcam Cat Number: M1248-250) gradient for a total volume of 12 mL (Fig. S6F). The gradient was top-loaded and then centrifuged at 50,000 × *g* for 1 h using a SW-41Ti rotor in an Optima XE-100 Ultracentrifuge. Twenty-four 500 μL fractions were collected from the top of the gradient and labeled as (starting from the top) 1a, 1b, 2a, 2b, etc., until 12b (the bottom fraction). Fractions of interest were diluted fivefold, re-centrifuged at 100,000 × *g* for 1 h, and resuspended in lysis buffer and protease inhibitor to remove potentially contaminating iodixanol from the Optiprep.

### Streptavidin co-immunoprecipitation

Fractions of interest from both *TgPDIA3-TID-3HA-GEEL* and *FBXO14-TID* were lysed in RIPA buffer (150 mM NaCl, 0.1% SDS, 0.5% sodium deoxycholate, and 1% NP-40 in 50 mM HEPES, pH 7.5) as previously described (115) and incubated with streptavidin magnetic beads for 1 h at RT. Proteins were eluted from the beads using a buffer with excess biotin (20 mM biotin, 1% SDS, and 25 mM Tris, pH 7.4) at 75**°**C for 30 min. Co-IP eluates were sent to the University of Nebraska Proteomics & Metabolomics Facility for LC-MS/MS analysis. Two biological replicates each for S4 and the combined ER gradient fractions 6a, 6b, 7a, and 7b were sent. The results were analyzed using Scaffold to determine those proteins enriched in the ER fractions in the TgPDIA3-TurboID cell line compared to S4 in FBXO14 cytosolic controls.

### Microneme secretion assays

Microneme secretion assays were conducted as previously described (108). Freshly egressed parasites were collected, filtered through an 8-µm nuclepore membrane, and resuspended in invasion media (20 mM HEPES in DMEM-HG) at 8 × 10^8^ parasites/mL and stored on ice. Fifty microliters of parasite suspension was aliquoted for a whole cell control and lysed using CelLytic M (Sigma Cat Number: C2978) with 1 µL DNase I (Thermo Cat Number: EN0521) and 1 µL RNase A (Thermo Cat Number: 19101), An aliquot of 100 µL of the parasite suspension was added to 100 µL of invasion media or invasion media containing 2× inducers (either 2 µM ionomycin or equal volume of DMSO). Suspensions were incubated either for 30 min at 37°C for constitutive secretion or for 3 min at 37°C with inducers or controls for induced secretion. After the incubation, the suspension was immediately placed in ice and centrifuged at 1,000 × *g* at 4°C for 5 min. An aliquot of 180 µL of the supernatant was placed in a fresh tube and centrifuged again to remove any remaining parasites, and 150 µL of the supernatant was placed in a fresh tube. A portion of the secretions was boiled with 4× Laemmli for 5 min. Western blots were conducted as described above. Blots were probed with rabbit αMIC2 at 1:1,000 (from Vern Carruthers) and mouse αGRA1 at 1:1,000 (generated in-house) to compare MIC2 secretion to the constitutively secreted GRA1 (108). Protein band intensity was measured using Image Studio.

### Microneme and rhoptry maturation assay

Microneme maturation assays were conducted as previously described with some modifications (108). Freshly egressed parasites were collected from wild-type and mutant strains either preincubated with or without ATc. Parasites were filtered through a 5-µm nuclepore membrane and lysed using CelLytic M. Protein concentrations were measured using BCA and evenly loaded into a 10% SDS-PAGE gel for subsequent western blotting.

Western blots and intracellular immunofluorescence assays were performed in *iΔTgPDIA3* and *TatiΔku80* parasites. Antibodies staining for different secreted proteins, including mouse αROP1 at 1:1,000 (from BEI resources), mouse αROP7 at 1:2,000 (from Peter Bradley), rabbit αMIC2, and mouse αMIC3 at 1:1,000 (from BEI resources), were utilized to probe the localization of different rhoptry and microneme proteins in knocked-down parasites (incubated with ATc for 48 h) compared to control parasites (0 h ATc and *TatiΔku80* parental) (108).

### Calcium measurements

For measuring SERCA activity, freshly lysed extracellular tachyzoites were filtered through an 8-µm nuclepore membrane, washed with BAG, and resuspended at a concentration of 1 × 10^9^ parasites/mL in a loading buffer with 20 µM Mag-Fluo-4 AM (CAT: 20401), 0.02% pluronic F127 (Thermo Cat Number: 20052), and 100 µg/mL BSA, in HEPES-buffered saline (HBS) (135 mM NaCl, 5.9 mM KCl, 11.6 mM HEPES, 1.5 mM CaCl_2_, 11.5 mM glucose, 1.2 mM MgCl_2_, pH 7.3) (46, 66). Parasites were incubated with the indicator for 1 h with shaking at RT and protected from light. After loading, parasites were washed twice with cytosol-like media (CLM) (1 mM EGTA, 20 mM PIPES, 20 mM NaCl, 140 mM KCl, pH 7.0) and permeabilized with 100 µM digitonin for 5 min. Parasites were washed twice and resuspended in CLM at 1 × 10^9^ parasites/mL and stored on ice. Fluorescence measurements of 2 × 10^7^ parasites were taken using a Hitachi F-7000 Fluorescence Spectrophotometer using Mag-Fluo-4 conditions for excitation (495) and emission (528). Free Ca²^+^ was clamped at 220 nM using an EGTA/Ca²^+^ buffering system (total CaCl_2_ added: 375 µM, calculated with MaxChelator: https://somapp.ucdmc.ucdavis.edu/pharmacology/bers/maxchelator/), together with 125 µM MgATP (SERCA substrate) and 1 µM thapsigargin (SERCA inhibitor), to characterize changes in ER calcium sequestration and SERCA function.

For measurement with GECI expressing parasites (65), freshly egressed GCaMP6f-mScarlet expressing parasites were utilized, and for mitochondrial calcium measurements, force-lysed intracellular GCaMP6f-SOD2 mutants were utilized. For all GECI experiments, parasites were collected, washed with BAG, resuspended at 1 × 10^9^ parasites/mL, and kept on ice. Fluorescence measurements were done using a Hitachi F-7000 Fluorescence Spectrophotometer using GCaMP6f conditions for excitation (485) and emission (509), and excitation (569) and emission (594) for mScarlet (65).

Final concentrations of reagents used during fluorometry experiments were as follows: 40 µM GPN, 1 µM thapsigargin, 100 µM zaprinast, 125 µM MgATP, 1 µM ionomycin, and 1.8 mM calcium (for non-permeabilized cells).

### Mitochondrial membrane potential measurements

Mitochondrial membrane potential was measured using the lipophilic cationic dye tetramethylrhodamine ethyl ester (TMRE), as previously described (116), with some modifications. Parasites were incubated at 37°C for 30 min under one of the following conditions: (i) 250 nM TMRE, (ii) 250 nM TMRE plus 5 µM FCCP, or (iii) DMSO (control), all prepared in BAG. After the incubation, parasites were pelleted and washed with BAG, except for the FCCP condition, which was washed with BAG containing 5 µM FCCP. Parasites were then resuspended at a concentration of 1 × 10^8^ parasites/mL in the corresponding buffer; 100 µL of parasite suspension was added to each well of a 96-well plate in triplicate and imaged on a BioTek Synergy H1 Hybrid Reader at excitation 520/25 nm and emission 590/35 nm.

### Ultrastructure expansion microscopy

Expansion microscopy was conducted with intracellular parasites as previously described (117). In brief, parasites grown on an HFF monolayer on coverslips were fixed 20 hpi, with 4% Paraformaldehyde (PFA) at 37°C for 20 min, followed by protein crosslinking prevention with formaldehyde and acrylamide. After 24 h, the cells were transferred to sodium acrylate gels (19% sodium acrylate, 10% acrylamide, 0.1% N,N′-methylenbisacrylamide, in PBS [pH 7.4], TEMED, and APS), and gels were transferred to a denaturing buffer (200 mM SDS, 200 mM NaCl, 50 mM Tris in water, pH 9.0) and heated at 90°C for 90 min. Gels were expanded through three 30-min incubations in water, then shrunk with PBS (pH 7.4) before blocking with 3% BSA in PBS-T. Samples were incubated overnight with primary antibodies in 3% BSA-PBS: rabbit αMIC2 (1:200; gift from Vern Carruthers), mouse αTgPDI1 (1:300; generated in-house), and guinea pig αSERCA (1:100; generated in-house). After three washes in PBS-T, gels were incubated with secondary antibodies and NHS-ester 405 (ThermoFisher) in PBS for 2.5 h, followed by three additional 30-min rounds of expansion in water. For imaging, gels were transferred to CellVis dishes (Thermo Cat Number: NC1129240) freshly coated with poly-D-lysine to minimize drift or stored at 4°C in 0.2% (wt/vol) propyl gallate for up to 48 h. Imaging was performed on a Zeiss LSM 980 using Airyscan 2, and 3D renderings were generated with IMARIS software.

### Protein identification with LC-MS/MS 

Samples were submitted to the Proteomics and Metabolomics Facility, Nebraska Center for Biotechnology, University of Nebraska-Lincoln, and analyzed for mass spectrometry (MS), as previously described (118). Briefly, an aliquot of 37.5 µL of the sample was added to 12.5 µL of 4× reducing NuPAGE LDS gel (Thermo Fisher Scientific, Waltham, MA) sample buffer at 5 mM dithiothreitol (DTT) and incubated at 95°C for 10 min. The samples were loaded and run on a Bolt 12% Bis-Tris-Plus gel (Thermo Fisher Scientific) in MES SDS running buffer to clean them and concentrate the proteins at the top of the gel. The gel was then fixed in methanol:acetic acid:water (40:10:50) and stained with Colloidal Coomassie blue G-250. The gel containing proteins was excised and destained in 50% acetonitrile (ACN) and 50 mM ammonium bicarbonate. The proteins were reduced in 100 mM ammonium bicarbonate with DTT at 10 mM. The reducing buffer was removed, and proteins were alkylated with iodoacetamide at 10 mM. Proteins were digested with 250 ng of trypsin overnight at 37°C. Peptides were extracted from the gel pieces, dried down, and re-dissolved in 5% acetonitrile, 0.2% formic acid. Each digest was run by nanoLC-MS/MS using a 2 h gradient on a Waters CSH 0.075 mm × 250 mm C18 column (Waters Corp, Milford, MA) feeding into a Thermo Orbitrap Eclipse mass spectrometer.

All MS/MS samples were analyzed using Mascot (Matrix Science, London, UK; version 2.7). Mascot was set up to search the cRAP_20150130.fasta (125 entries) and ToxoDB-59_TgondiiGT1_AnnotatedProteins_20221003 (8,460 sequences), assuming the digestion enzyme trypsin. Mascot was searched with a fragment ion mass tolerance of 0.060 Da and a parent ion tolerance of 15.0 PPM. Carbamidomethyl of cysteine was set as a fixed modification. Deamidation of asparagine and glutamine, oxidation of methionine was specified in Mascot as variable modifications. Scaffold (version Scaffold_5.2.2; Proteome Software Inc., Portland, OR) was used to validate LC-MS/MS-based peptide and protein identifications.

Hits were identified with 99% protein threshold, 95% peptide threshold, and a minimum peptide number of 2. Statistical analysis was conducted using Fisher’s exact test with Benjamini-Hochberg multiple test corrections to identify significantly enriched proteins.

### Statistical analysis

Experimental data were expressed as the mean with standard error (SEM) from at least three biological replicates unless otherwise indicated. Statistical analyses were completed using GraphPad PRISM and R. Student’s *t*-test, one-way ANOVA, two-way ANOVA, linear regression, and linear mixed model were used when necessary and are indicated in figure legends. A *P*-value of <0.05 was considered statistically significant. For all LC-MS/MS analyses, in Scaffold, protein thresholds were set to 95%, a minimum peptide number was set to 2, and the peptide threshold was set to 99%. Fisher’s exact test (with Benjamini-Hochberg multiple testing corrections) was used to calculate statistical significance. Minimum values were set to 0.0001 to avoid infinity.

## Data Availability

The proteomic data generated in this study have been deposited in the PRIDE repository (119) under the following identifiers: SERCA-HA DVSF HA-IP, PXD063659; streptavidin-IP, PXD063619; and DVSF αTgPDIA3-IP, PXD063615. All other data generated are included in the main figures, tables, and supplemental material.

## SUPPLEMENTAL MATERIAL

Supplemental Information mbio.03124-25-s0001.pdf Supplemental tables and figures.

Supplemental Tables mbio.03124-25-s0002.xlsx Tables S4, S5, and S7.

Video S1 mbio.03124-25-s0003.mp4 U-ExM of *T. gondii* ER and micronemes.

ASM does not own the copyrights to Supplemental Material that may be linked to, or accessed through, an article. The authors have granted ASM a non-exclusive, world-wide license to publish the Supplemental Material files. Please contact the corresponding author directly for reuse.
