# Supplementary material for: A protein disulfide isomerase coordinates redox homeostasis and ER calcium regulation for optimal lytic cycle progression in Toxoplasma gondii
Source: mBio. 2026 Apr 1;17(5):e03124-25. doi: 10.1128/mbio.03124-25 (PMC13170237; doi:10.1128/mbio.03124-25)
Supplement: Supplemental Information — Supplemental tables and figures. [file mbio.03124-25-s0001.pdf]

A Protein Disulfide Isomerase Coordinates Redox Homeostasis and ER Calcium Regulation for Optimal Lytic Cycle Progression in *Toxoplasma gondii*

Katherine E. Moen and Silvia N J Moreno

Supplementary Information

Supplementary Tables

Table S1. *T. gondii* predicted PDIs

Gene ID	Annotation	Fitness score	# of TM	LOPIT predicted	Active site	Predicted Functions
TGME49_271760	SerRS2	-2.84	1	ER	CXXC	glycosyl-transferase activity
TGME49_258826	hypothetical	-3.88	1	ER	CXXC	
TGME49_201800	hypothetical	-4.01	0	mitochondrion	SXXC	
TGME49_232410	*PDI	0.99	0	cytoskeleton		PDR activity
TGME49_211680	PDI	-5.65	0	**ER	CXXC	Ca^{2+} ion binding glycosyl-transferase activity PDI activity
TGME49_312110	Atrx1	-4.16	0	apicoplast	CXXS	PDR activity
TGME49_224060	*Trx	-1.29	0	Golgi	CXXC	glycosyl-transferase activity
TGME49_238040	*PDI	-3.48	0	ER	CXXC	PDI activity
TGME49_247350	*Trx	0.8	4	ER	CXXC	PDI activity
TGME49_225790	*PDI	1.98	0	cytoskeleton		
TGME49_216510	*Trx	-4.8	1	mitochondrion	CXXC	
TGME49_249270	*PDI	-4.56	0	**ER	CXXC	PDI activity
TGME49_255480	*Trx	-0.61	1	-	CXXC	PDI activity
TGME49_209950	*Trx	-5.2	0	**ER	CXXC	
TGME49_291810	*Trx	-0.76	0	-	CXXS	
TGME49_247660	*Trx	1.04	0	-	CXXC	
TGME49_266620	CTRX1	1.28	0	ER	CXXC	
TGME49_232050	*DnaJ	-4.16	2	ER	CXXC	DNAJ domain
TGME49_217710	*DnaJ	-0.01	0	-	CXXC	DNAJ domain
TGME49_218470	*PDI	0.61	0	-	CXXC	PDI activity
TGME49_270120	TLP1	-1.61	0	mitochondrion	CXXC	
TGME49_300150	hypothetical	-1.1	0	nucleus	CXXS	UAS
TGME49_204480	*Trx	-3.49	0	apicoplast	CXXC	DNAJ domain
TGME49_277790	hyp	-2.65	0	nucleus	SXXC	PDR activity
TGME49_289180	*Trx	1.28	0	mitochondrion	CXXC	PDR activity
TGME49_225060	nucleoredoxin	-0.9	0	nucleus	CXXC	PUB domain

Supplementary Table S1. *T. gondii* predicted PDIs. List of all *T. gondii* genes with the Interpro domain prediction "thioredoxin-like domain superfamily". Genes annotated as PDIs were automatically included and genes that were specifically identified as other Trx-family members: glutaredoxin, peroxiredoxin, thioredoxin, or glutathione S-transferase, were excluded. Columns include information for gene ID, predicted name (* denotes "putative", "family protein", or "domain-containing protein" as a part of the name), CRISPR fitness score [9], number of predicted transmembrane domains, LOPIT predicted localization [10] (** denotes localization by IFA for PDIs in this study), active site motif, and predicted functions based on GO terms or Interpro including PDI activity, protein disulfide reductase (PDR) activity, calcium ion binding, DNAJ domain containing proteins, and glycosyltransferase activity.

Table S2. Phylogenetic sequences used for Fig S1A

Phylogeny	Organism	Genebank/VEuPathDB	Reference
TGGT1_211680 (TgPDIA3)	*Toxoplasma gondii*	TGGT1_211680	this work
	Hammondia hammondi	HHA_211680	predicted ortholog
	Besnoitia besnoiti	BESB_016710	predicted ortholog
	Cystospora suis	CSUI_002988	predicted ortholog
	Sarcocystis neurona	SN3_02200010	predicted ortholog
	Cyclospora cayetanensis	cyc_03227	predicted ortholog
	Plasmodium knowlesi	PKNH_1322000	predicted ortholog
	Plasmodium vivax	PVP01_0506500	[1]
	Saccharomyces cerevisiae	YCL043C	[2-4]
	Sporisorium reilianum	sr10870	predicted ortholog
	Leishmania donovani	LdBPK_367280.1	[5]
	Trypanosoma cruzi	TcCLB.507611.370	predicted ortholog
	Trypanosoma brucei	Tb11.v5.0668	predicted ortholog
PDIA3	*Homo sapiens*	P30101	[6]
	Mus musculus	P27773	[7]
PDIA4	*Homo sapiens*	P13667	[8]
	Mus musculus	P08003	[11]
TGGT1_249270 Putative Protein disulfide isomerase related protein (PDIA6)	*Toxoplasma gondii*	TGGT1_249270	this work
	Hammondia hammondi	HHA_249270	predicted ortholog
Protein disulfide isomerase (PDIA6)	*Homo sapiens*	ENSG00000143870	[12]
	Mus musculus	ENSMUSG0000002057 1	[13]
Thioredoxin-related transmembrane protein 1 (TMX1)	*Homo sapiens*	Q9H3N1	VEuPathDB
	Mus musculus	Q8VBT0	VEuPathDB
Thioredoxin domain containing protein (TRX1)	*Toxoplasma gondii*	TGGT1_247350	ToxoDB
	Hammondia hammondi	HHA_247350	ToxoDB
Conserved Archaeal Protein	*Hyperthermus butylicus*	A2BLB3	Uniprot PDI
Putative thioredoxin (TgERDJ3A)	*Toxoplasma gondii*	TGGT1_209950	ToxoDB
	Hammondia hammondi	HHA_209950	ToxoDB

Supplementary Table S2. Sequences used for the phylogenetic analysis shown in Fig. S1A, including organism, gene ID, and sequence retrieval method. BLAST predictions were conducted on VEUpathDB.org. Outgroups include other PDI family member proteins.

Table S3. List of Primers used

Cell Line	Primer name	Sequence
Recombinant TgPDIA3	211680 gene Fwd	atgcgagccgggttttcgtttgctctgttggcagtcggcc
	211680 gene Rev	ttacagttcttcacccttgtcgtccttc
	211680 gene with homology Fwd	tcaccatcaccgagccgggttttcgtttg
	211680 gene with homology Rev	gagtccaagcttacagttcttcacccttgtcg
	pQE80L with 211680 homology Fwd	agaactgtaagcttggactcctgttgatag
	pQE80L with 211680 homology Rev	acccggctcggtgatggtgatggtgatg
Recombinant TgPDIA6	249270 gene Fwd	atggcgttcacaggcgcg
	249270 gene Rev	tcaaagttcatctttcggaagttcttcatctttg
	249270 gene with homology Fwd	tcaccatcacgcgttcacaggcgcgcgc
	249270 gene with homology Rev	gagtccaagctcaaagttcatctttcggaagttcttcatctttgccatcc
	pQE80L with 249270 homology Fwd	tgaactttgagcttggactcctgttgatag
	pQE80L with 249270 homology Rev	ctgtgaacgcgtgatggtgatggtgatg
iΔTgPDIA3-3Ty	211680 PI3Ty Fwd	tcctccgtctccgtcgtacccaggaagttttccctgtcggcatatgcgtgactttcccgc
	211680 PI3Ty Rev	ggccgactgccaacagagcaaacgaaaacccggctcgcatgtccaggggatcctgattg
	211680 N-terminal gRNA Fwd	gctttgtctcgcttagagcctgttttagagctagaaatagcaag
	211680 N-terminal validation Fwd	acacctccactgtttccggaaca
iΔTgPDIA6-3Ty	249270 PI3Ty Fwd	tgtcgactctttcttcgctgctgacgagcacaccgcgttccatatgcgtgactttcccgc
	249270 PI3Ty Rev	gaggtgcaccgcgccgcatgcgcgcgcctgtgaacgccatgtccaggggatcctgattg
	249270 N-terminal gRNA Fwd	gtcccgcgcctcgaaaaattcgttttagagctagaaatagcaag
	249270 N-terminal validation Fwd	cccttcctaccttctgcatttccca
pLIC3-HA-KDEL plasmid	pLIC-3HA-KDEL Fwd	gaactttaacccgggcatatgtagaaaagttgtaacg
	pLIC-3HA-KDEL Rev	atctttggcataatctggaacatcgtaaggatacg
TgPDIA6-3HA-KDEL	249270-pLIC-3HA-KDEL homology Fwd	tcgcgtgcgtgggatggcaaagatgaagaacttccgattggaagtggaggacgggaattc
	249270-pLIC-3HA-KDEL homology Fwd	cccatctctctgtcggaggctgtctgcaggacagcaagaattgtgttaaccggttcgact
iΔTgPDIA3 (no tag)	211680 PI no tag Fwd	ggccgactgccaacagagcaaacgaaaacccggctcgcatggttgaagacagacgaaagc
	211680 PI no tag Rev	gaggtgcaccgcgccgcatgcgcgcgcctgtgaacgccatggttgaagacagacgaaagc
pLIC3-HA-GEEL plasmid	pLIC-3HA-GEEL Fwd	gaactttaacccgggcatatgtagaaaagttgta
	pLIC-3HA-GEEL Rev	ttcaccggcataatctggaacatcgtaa
TgPDIA3-3HA-GEEL	6803HACFwd	aagccgctcaagaaggacgacaagggtgaagaactgattggaagtggaggacgggaattc
	6803HACRev	gggttatattgacggaaacatgagccaaacagggcaagaattgtgttaaccggttcgact

	211680 C-term gRNA	ggtacatgagcggttgaaacagttttagagctagaaatagcaag
TurboID-3HA-GEEL plasmid	TurboID-3HA-GEEL Fwd	gaactttaacccgggcatatgtagaaaagttgtaa
	TurboID-3HA-GEEL Rev	ttcacccttttcggcagaccg
TgPDIA3-TBID-GEEL	211680 TurboID Fwd	aagcacggttccaagccgctcaagaaggacgacaagtacccgtacgacgtcccggactac
	211680 TurboID Rev	gggttatattgacggaaacatgagccaaacaggccctcgggggggcaagaattgtgttaa
Recombinant RFP	RFP sequence Fwd	tcaccatcacatggcgcctagggtgagc
	RFP sequence Rev	gagtccaagccttgtacagctcgtccatgc
	pQE80L with RFP homology Fwd	gctgtacaaggcttggactcctgttgatag
	pQE80L with RFP homology Rev	taggcgccatgtgatggtgatggtgatg
	RFP validation rev	ccgcgcatcttcaccttgtagatca
Recombinant GFP	GFP sequence Fwd	tcaccatcacatggtgagcaagggcgag
	GFP sequence Rev	gagtccaagcttacttgtacagctcgtccac
	pQE80L with GFP homology Fwd	gtacaagtaagcttggactcctgttgatag
	pQE80L with GFP homology Rev	tgctcaccatgtgatggtgatggtgatg
	GFP validation Fwd	gggcatggcggacttgaagaa
Recombinant GRA1 for antibody	GRA1 gene Fwd	tcaccatcacatggtgcgtgtgagcgct
	GRA1 gene Rev	gagtccaagcttactctctctctcctgttaggaacc
	pQE80L with GRA1 homology Fwd	gagagagtaagcttggactcctgttgatag
	pQE80L with GRA1 homology Rev	cacgcaccatgtgatggtgatggtgatg
	GRA1 validation Rev	tcgctgtacgatccatctgaagctttaat
TgERDJ3A-3HA-KDEL	209950 C-3HA Fwd	aaggaagaaacagagaagaaggagaaggcagacaagattggaagtggaggacgggaa
	209950 C-3HA Rev	aaacgtacgacggggagagacaggaaagggaacgcaagaattgtgttaaccggttcgact
	209950 C-3HA C-Val F	ttcaaggttgtgccatcgagcaaga
	209950 C-Cas9 Fwd	gacatctaccggatctctgtggttttagagctagaaatagcaag
iΔTgERDJ3A-3Ty	209950 N-Cas9 Fwd	gtgtggcggtgggggtcgctggttttagagctagaaatagcaag
	209950 PI3Ty Fwd	actgtcccctcctcctcccctcccccctcctcccccgcccatatgcgtgactttcccgc
	209950 PI3Ty Rev	agacgagaggcaagaacgcgcacacacgcagaggcgccatgtccaggggatcctgattg
	209950 N-val Rev	gaacttctggaccttcccgtccc
iΔTgERO1-3Ty	ERO1 N-gRNA	gctcaggcgtgaccagaccaggttttagagctagaaatagcaag
	ERO1 PI3Ty Fwd	cattgaaaagccggcccttgggaggtcgtttcgaattcagcatatgcgtgactttcccgc
	ERO1 PI3Ty Rev	gacgccttctccttatttccctatactgagatccttccatgtccaggggatcctgattg
	ERO1 N-val Rev	aaccgaagacaacgagcgaaaatcc
Other validation primers	(AJ11) pLIC-3HA val Rev	ggatagccagcgtagtccggg
	(AJ121) pQE-80L seq Rev	cttccttagctcctgaaaatctcgcc

Supplementary Tables S4, S5, and S7 contain the proteomics results for DVSF αTgPDIA3-IP, TgPDIA3TID αStreptavidin-IP, and DVSF SERCA-3HA αHA-IP respectively, and are listed in the proteomics results excel file.

Table S6: Proteins enriched in both TgPDIA3-TID ER and TgPDIA3-IP +DVSF fractions

Gene ID	Gene product	L2FC +DVSF	L2FC TID ER
TGGT1_319560	MIC3	9.39	6.41
TGGT1_201780	MIC2	7.64	3.91
TGGT1_292020	GCC2/GCC3	8.37	4.39
TGGT1_300350	cysteine desulfurase/selenocysteine lyase family PLP dependent transferase superfamily protein	10.23	4.32
TGGT1_248880	GTPase RAB7	2.00	4.32
TGGT1_247350	thioredoxin domain-containing protein	9.76	3.91
TGGT1_253900	parasite porphobilinogen synthase PBGS	8.08	3.91

Supplementary Table S6. Proteins significantly enriched in both TgPDIA3 +DVSF and TgPDIA3-TID ER fraction. Including gene ID, protein product description, and Log_2 ratio of the fold change (L2FC) for both TgPDIA3 +DVSF and TgPDIA3-TID ER fraction.

Supplementary Figures

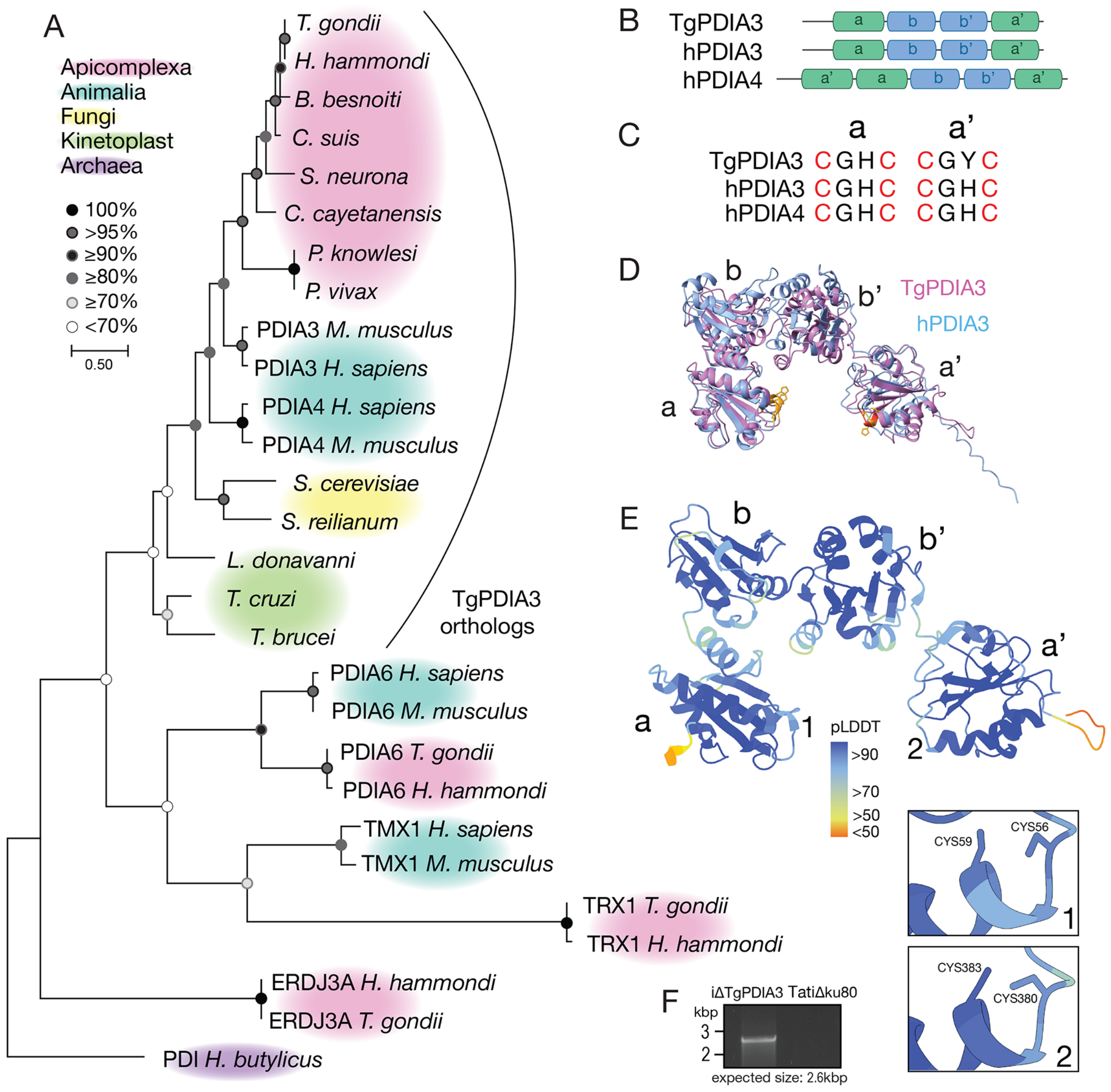

Figure S1. TgPDIA3 is structurally similar to HsPDIA3. A) Phylogenetic analysis of TgPDIA3 and predicted putative orthologs, including both mammalian PDIA3 and PDIA4, and PDI family outgroups. The sequences used are shown in **Table S2**. B) Organization of the PDI conserved **a**, **b**, **b’** and **a’** domains in TgPDIA3, HsPDIA3, and HsPDIA4. C) CXXC motifs in the **a** and **a’** domains of TgPDIA3, HsPDIA3, and HsPDIA4. D) Overlay of the TgPDIA3 and PDIA3 AlphaFold v2.0 predicted models. E) AlphaFold v2.0 model of TgPDIA3 indicating the two CXXC active sites (1 and 2), and the four globular domains, with average pLDDT scores: a, 92.42; b, 93.54; b′, 94.15; a′, 94.72. F) PCR validation of *iΔTgPDIA3-3Ty* mutant.

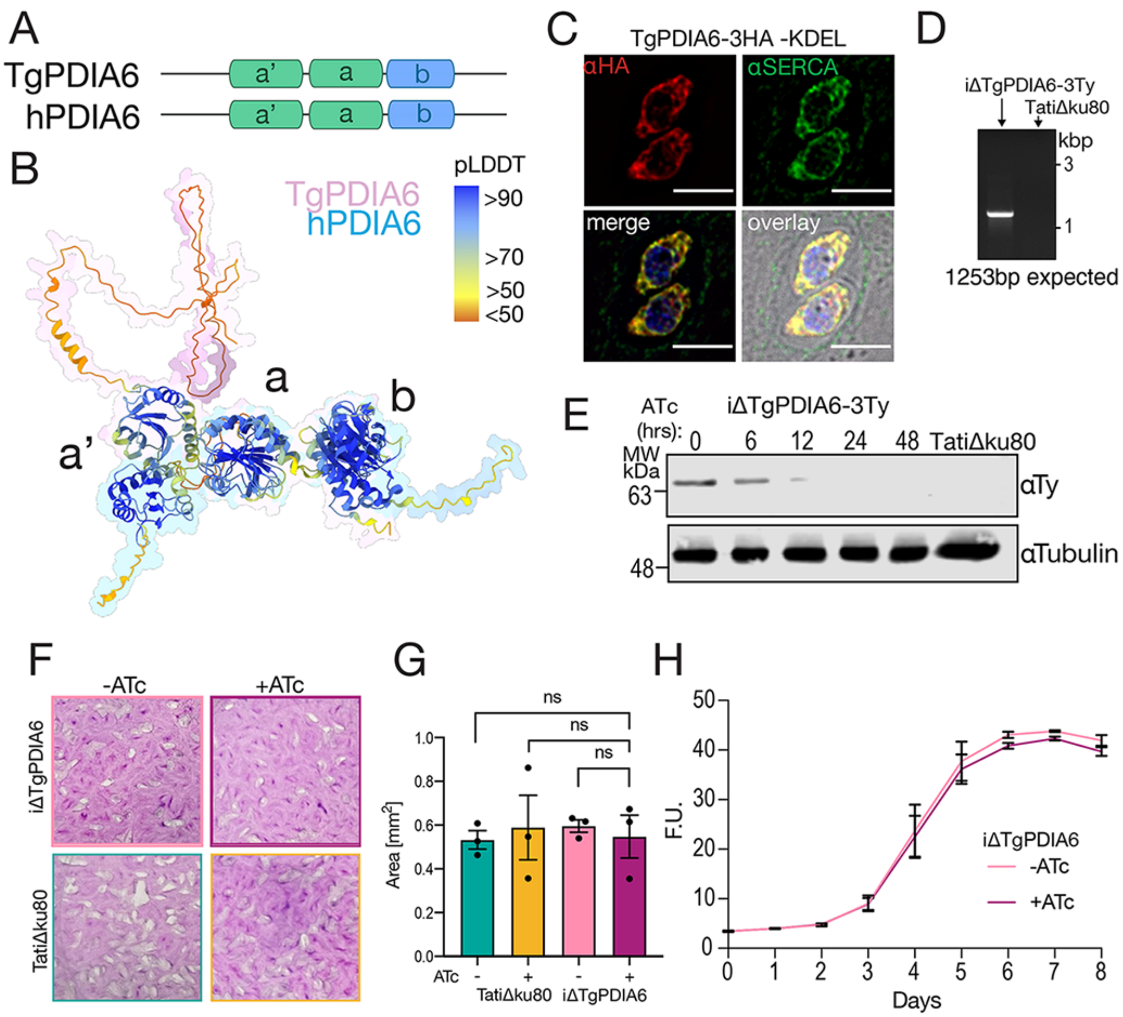

Figure S2. **TgPDIA6 is dispensable for *T. gondii* growth.** A) Catalytic *a', a,* and non-catalytic *b* domains for TgPDIA6 and the human PDIA6. B) AlphaFold v2.0 model overlay with average pLDDT scores for the TgPDIA6 domains being **a'**: 87.79, **a**: 89.68, **b**: 92.14, and hPDIA6 domains being **a'**:91.95 **a**:92.12 **b**:93.74. C) Immunofluorescence assay of the *TgPDIA6-3HA-KDEL* mutant probed with rat αHA (1:25) and guinea pig αTgSERCA (1:500). D) PCR validation for the *iΔTgPDIA6-3Ty* clone. E) Western blot showing ATc downregulation of TgPDI6 after the addition of ATc in the *iΔTgPDIA6-3Ty* mutant. F) Representative plaque assay of the *iΔTgPDIA6-3Ty* mutant and *TatiΔku80* (control) incubated for 7 days ± ATc and G) Quantification of plaque size (mm^2) with statistical analysis performed using one-way ANOVA (n=3). H) Measurement of RFP fluorescence over 8 days of the *iΔTgPDIA6-RFP* mutant incubated ± ATc (n=3).

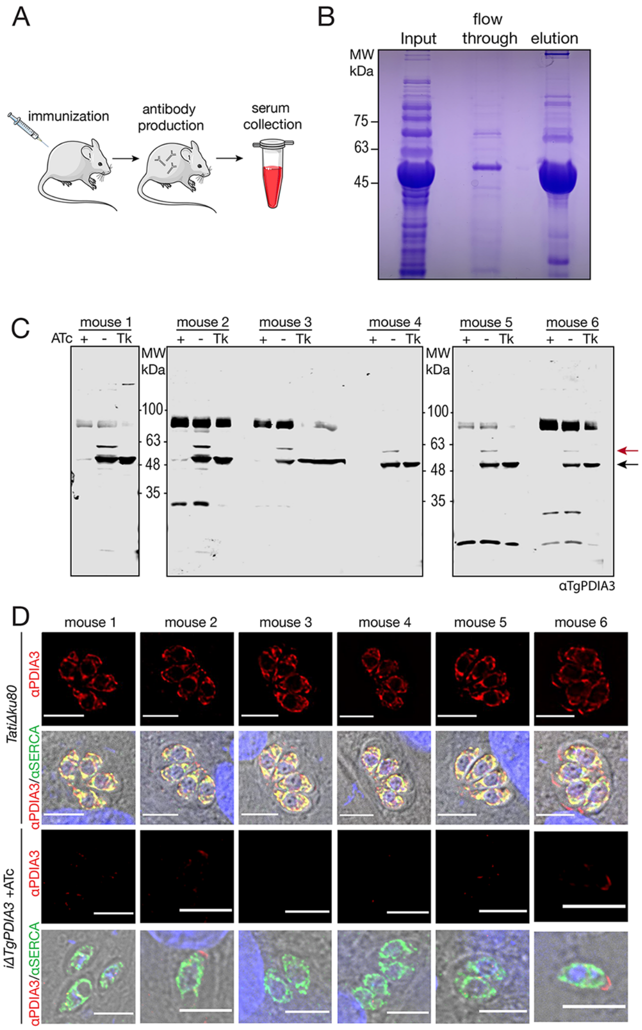

Figure S3. Generation of the αTgPDIA3 antibody A) cartoon depicting antibody production in mice. B) Coomassie-stained gel showing the purification of the TgPDIA3 protein. C) Western blot with *TatiΔku80* (Tk) and *iΔTgPDIA3*, (-ATc and +ATc) probed with the serum of 6 mice immunized with the TgPDIA3 antigen. The black arrow indicates the predicted band corresponding to TgPDIA3 after signal peptide and 3Ty tag removal and the red arrow indicates the predicted band of TgPDIA3-3Ty prior to signal peptide and 3Ty tag removal. D) Immunofluorescence assay of the *TatiΔku80* and *iΔTgPDIA3* (+ATc) mutant parasites probed with the serum from each of the 6 mice immunized with TgPDIA3 antigen (scale bar is 5 μm).

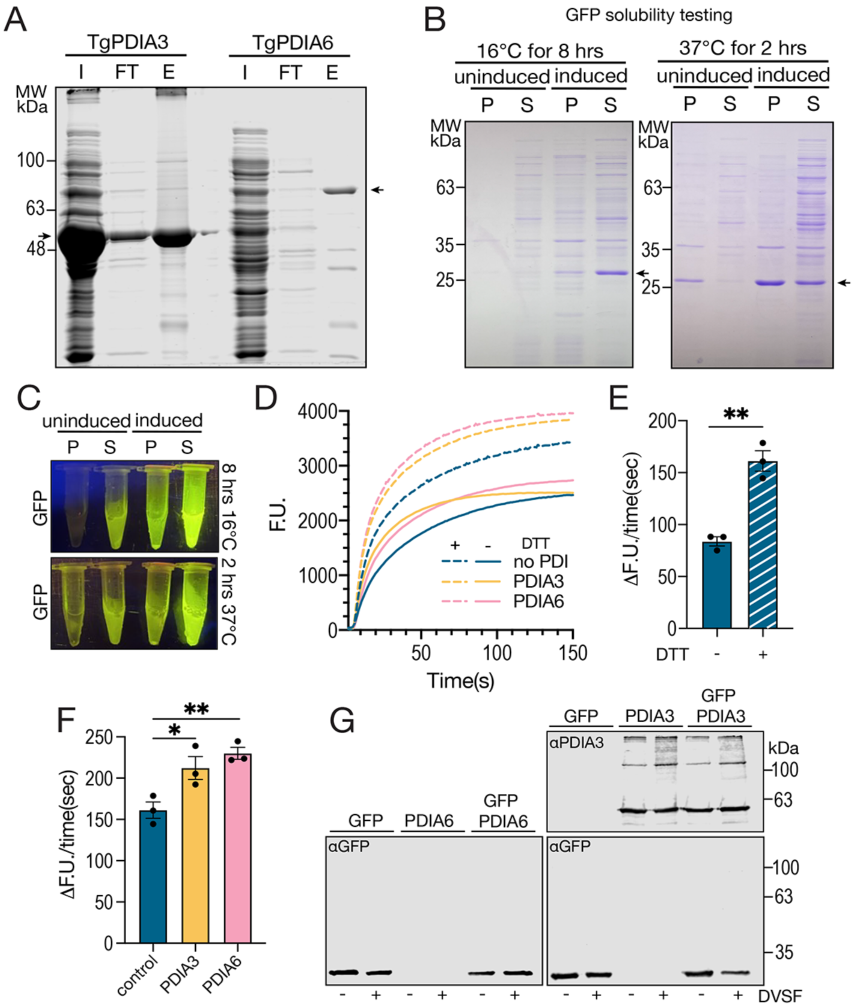

Figure S4. Generation of recombinant GFP, TgPDIA3 and TgPDI6. A) Coomassie-stained gel showing the protein purification of TgPDIA3 and TgPDIA6 (protein bands indicated by arrows). B) Coomassie-stained gel assessing the solubility of recombinant GFP (GFP protein band indicated with black arrows) (S = soluble, P = pellet). C) Tubes of recombinant GFP in each condition showing fluorescence in the induced soluble fraction. D) Average traces of 1 µM GFP fluorescence recovery in the absence of PDI (control) or in the presence of 5 µM TgPDIA3 or 2.5 µM TgPDIA6 and in the presence or absence of 1 mM DTT. E) Quantification of the initial 7-second slope of GFP fluorescence recovery in the presence or absence of 1 mM DTT. Statistical analysis was done with student's t-test (n=3). F) Quantification of the initial 7-second slope of GFP fluorescence recovery in the presence of 1 mM DTT and in the absence of PDI (control) or in the presence of 5 µM TgPDIA3 or 2.5 µM TgPDIA6. Statistical analysis was performed using one-way ANOVA (n = 3). G) Representative western blot of denatured GFP renatured with or without PDIA3 or PDIA6 and with or without DVSF.

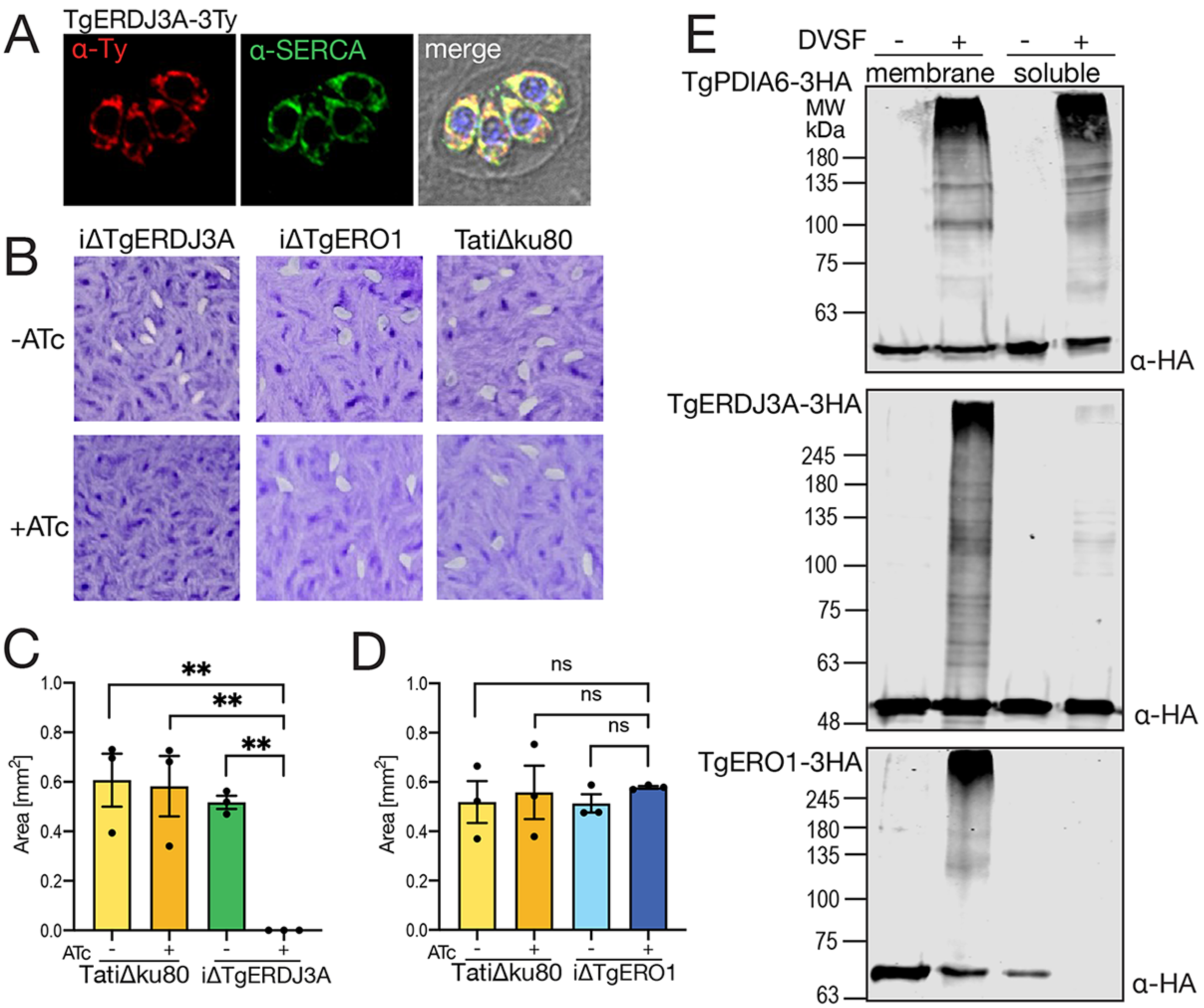

Figure S5. TgERO1 and TgERDJ3A are ER redox enzymes. A) Immunofluorescence assay of the *iΔTgERDJ3A-3Ty* mutant probed with αTy (red) and αSERCA (green). B) representative plaque assays of *iΔTgERO1-3Ty*, *iΔTgERDJ3A-3Ty* and *TatiΔku80* (control) lines incubated for 7 days ± ATc. Quantification of average plaque size (mm^2) for the *iΔTgERDJ3A-3Ty* (C) or *iΔTgERO1-3Ty* (D) mutants both including *TatiΔku80* (control) (n=3). E) Western blot of *iΔTgPDIA6-3HA*, *iΔTgERDJ3A-3HA*, and *iΔTgERO1-3HA* probed with αHA, incubated with or without DVSF for 30 min, showing either soluble proteins (right) or enriched membrane proteins (left). All quantifications were performed using one-way ANOVA for statistical analysis (n=3).

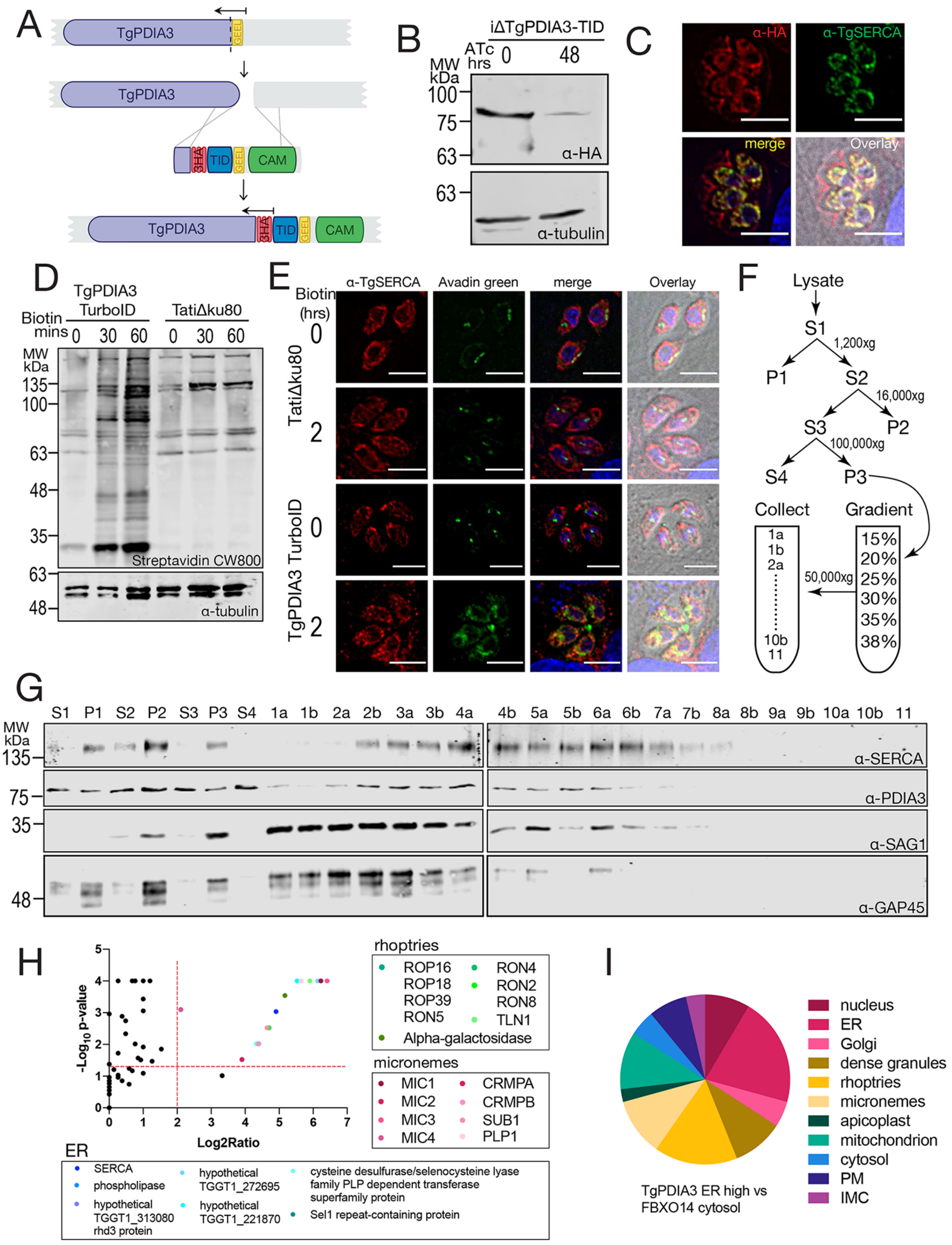

Figure S6. Mapping the Proximal Interactome of TgPDIA3-TID. A) Scheme showing the 3HA-TurboID cassette added to the C-terminus of *Tgpdia3,* including the GEEL retention signal downstream to the TURBO domain and a 3xHA tag. B) western blot validation of *TgPDIA3-TID-3HA-GEEL* tagging. C) IFA showing co-localization of *TgPDIA3-TID-3HA-GEEL* with TgSERCA used as ER marker. D) Western blot of *TgPDIA3-TID-3HA-GEEL*, or parental (*TatiΔku80*) cells incubated with 50 µM biotin for 0-, 30-, and 60-min. Streptavidin shows biotinylated proteins, and

tubulin was the loading control (scale bar, 5 μm). E) IFA showing the localization of biotinylated proteins in *TgPDIA3-3HA-TID-GEEL* parasites compared to *TatiΔku80* (WT), detected using Avidin green. F) scheme of the subcellular fractionation protocol. Details are in the Materials and Methods section. G) Western blots of the fractions obtained following the procedure shown in F, probed with αTgSERCA and αTgPDIA3 to detect ER-resident proteins, and with αSAG1 and αGAP45 to detect plasma membrane and inner membrane complex (IMC) proteins, respectively. H) Volcano plot of proteins enriched from the LC-MS/MS analysis. I) LOPIT [10]-predicted localization of proteins to the ER, microneme, or rhoptries. Fisher's exact test was used for statistical analysis (n=2). The complete list of enriched peptides is shown in the supplemental table: Proteomic Results: **Table S5** Streptavidin-IP.

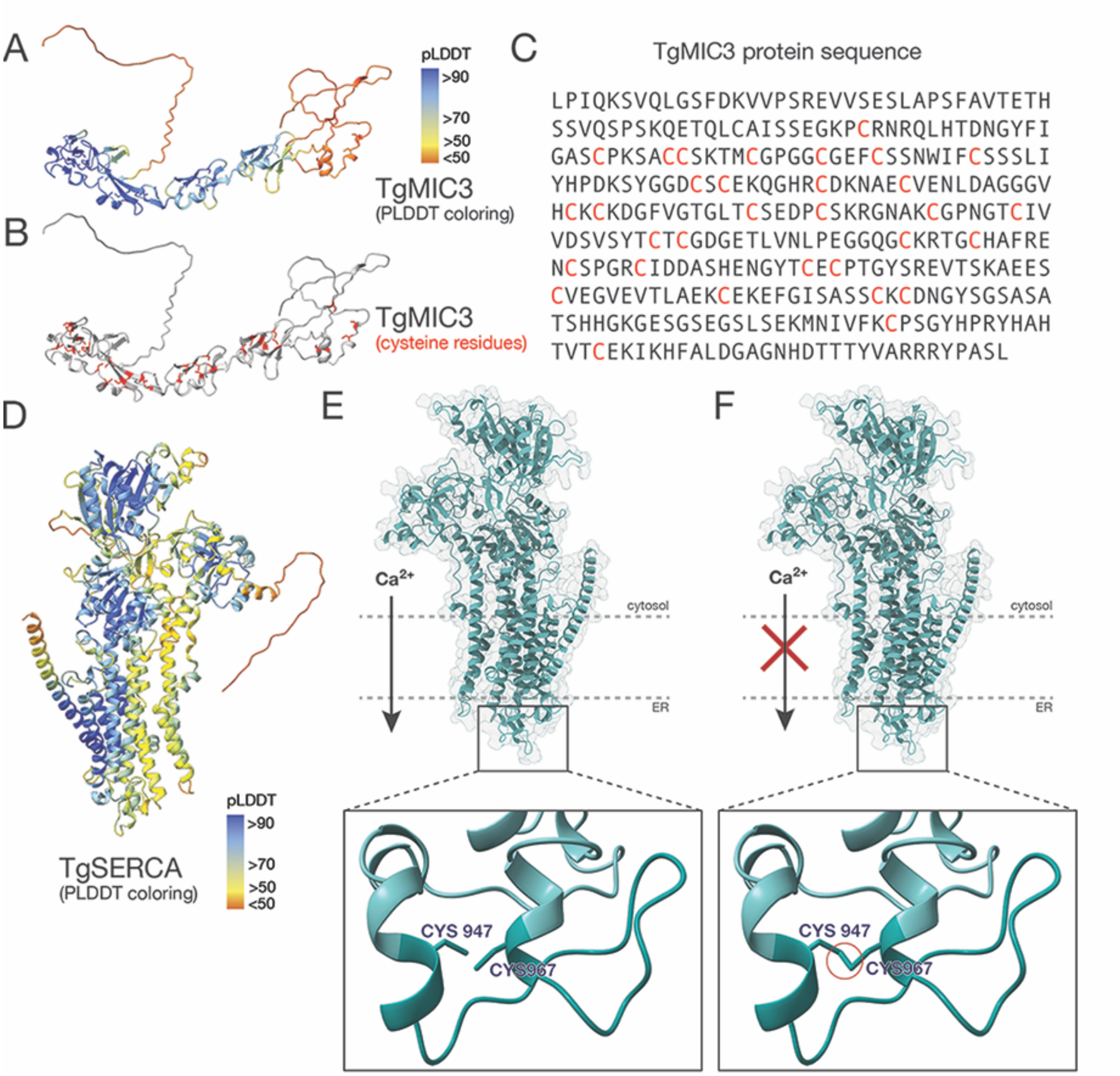

Figure S7. Cysteine residues in protein substrates. A-B) AlphaFold v2.0 model of TgMIC3 colored following the PLDDT scale (A) and highlighted cysteine residues in red (B). C) TgMIC3 protein sequence with the predicted signal peptide removed and with Cys residues highlighted in red. D) AlphaFold v2.0 model of TgSERCA with coloring following the PLDDT scale. E-F) AlphaFold v2.0 model of TgSERCA with its L4 loop including the cysteine residues predicted to form a disulfide bond for SERCA redox regulation (CYS 947 and CYS 967). E) active SERCA with a reduced L4 loop and F) inhibited SERCA with an oxidized L4.

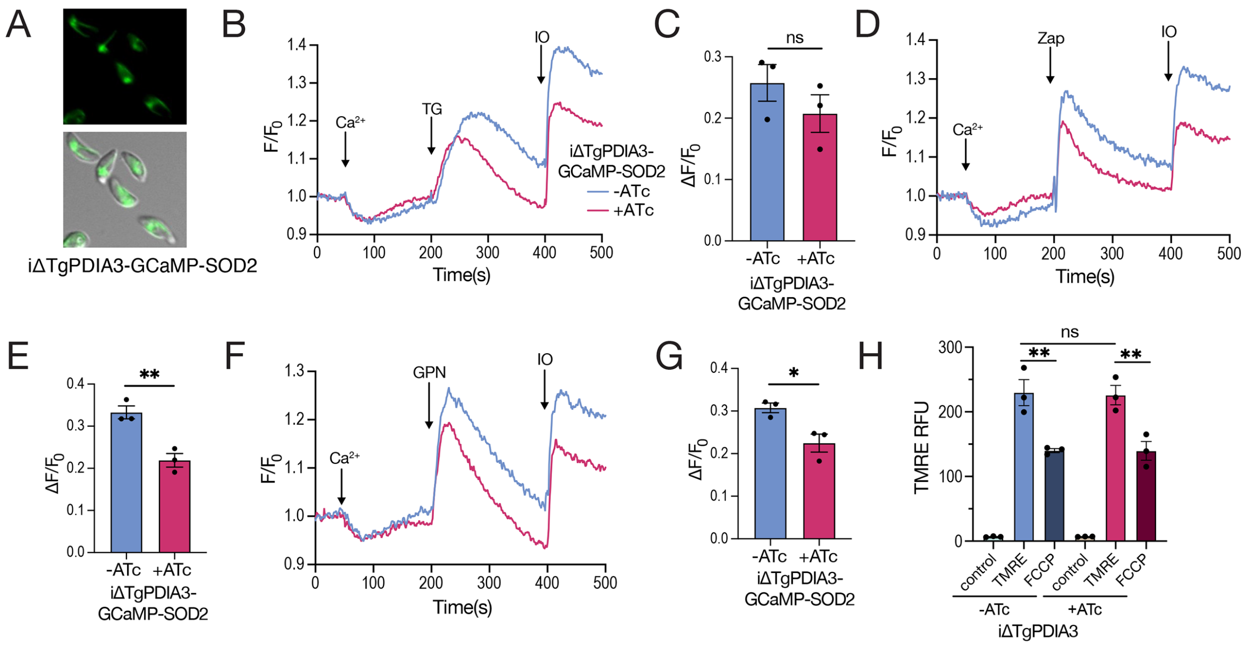

Figure S8. Calcium uptake by the mitochondrion of the *iΔTgPDIA3-GCaMP6f-SOD2* mutant. A) Image of live i*ΔTgPDIA3-GCaMP6-SOD2* mutant parasites. B) Normalized mitochondrial GCaMP6 fluorescence changes in response to 1 μM thapsigargin (TG) and 1 μM ionomycin (IO) in the presence of 1.8 mM extracellular Ca^{2+}; C) quantification of normalized fluorescence change after addition of TG. D) Normalized mitochondrial GCaMP6 fluorescence changes in response to 100 μM zaprinast (Zap) and 1 μM IO in the presence of 1.8 mM extracellular Ca^{2+}; E) quantification of normalized fluorescence change after addition of Zaprinast (Zap). F) Normalized mitochondrial GCaMP6 fluorescence changes in response to 40 μM GPN and 1 μM IO in the presence of 1.8 mM extracellular Ca^{2+}; G) quantification of normalized fluorescence change after addition of GPN. All the previous quantifications were analyzed using student's t-test for statistical significance (n=3). H) Mitochondrial membrane potential measurements of *iΔTgPDIA3* ± ATc using TMRE as described in the Materials and Methods section. TMRE shows mitochondrial membrane potential as fluorescence RFUs. Controls are parasites without TMRE (control). TMRE RFU indicate parasites loaded with TMRE, which are compared with parasites incubated with an uncoupler (FCCP) to show depolarization. The data was analyzed using one-way Anova for statistical significance (n=3).

Supplemental Video 1. U-ExM of *T. gondii* ER and micronemes. Representative IMARIS v9 surfaces created from ultrastructure expansion microscopy with immunofluorescence labeling of intracellular *TatiΔku80* tachyzoites probed with αTgPDIA3 (clear red) and αMIC2 (solid blue).

References

1. Mahajan B, Noiva R, Yadava A, Zheng H, Majam V, Mohan KV, et al. Protein disulfide isomerase assisted protein folding in malaria parasites. Int J Parasitol. 2006;36(9):1037-48. Epub 20060530. doi: 10.1016/j.ijpara.2006.04.012. PubMed PMID: 16806221.

2. Tachikawa H, Miura T, Katakura Y, Mizunaga T. Molecular structure of a yeast gene, PDI1, encoding protein disulfide isomerase that is essential for cell growth. J Biochem. 1991;110(2):306-13. doi: 10.1093/oxfordjournals.jbchem.a123576. PubMed PMID: 1761527.

3. Scherens B, Dubois E, Messenguy F. Determination of the sequence of the yeast YCL313 gene localized on chromosome III. Homology with the protein disulfide isomerase (PDI gene product) of other organisms. Yeast. 1991;7(2):185-93. doi: 10.1002/yea.320070212. PubMed PMID: 2063627.

4. LaMantia M, Miura T, Tachikawa H, Kaplan HA, Lennarz WJ, Mizunaga T. Glycosylation site binding protein and protein disulfide isomerase are identical and essential for cell viability in yeast. Proc Natl Acad Sci U S A. 1991;88(10):4453-7. doi: 10.1073/pnas.88.10.4453. PubMed PMID: 1840696; PubMed Central PMCID: PMCPMC51678.

5. Kushawaha PK, Gupta R, Tripathi CD, Sundar S, Dube A. Evaluation of Leishmania donovani protein disulfide isomerase as a potential immunogenic protein/vaccine candidate against visceral Leishmaniasis. PLoS One. 2012;7(4):e35670. Epub 20120423. doi: 10.1371/journal.pone.0035670. PubMed PMID: 22539989; PubMed Central PMCID: PMCPMC3335089.

6. Peaper DR, Wearsch PA, Cresswell P. Tapasin and ERp57 form a stable disulfide-linked dimer within the MHC class I peptide-loading complex. EMBO J. 2005;24(20):3613-23. Epub 20050929. doi: 10.1038/sj.emboj.7600814. PubMed PMID: 16193070; PubMed Central PMCID: PMCPMC1276702.

7. Celli CM, Jaiswal AK. Role of GRP58 in mitomycin C-induced DNA cross-linking. Cancer Res. 2003;63(18):6016-25. PubMed PMID: 14522930.

8. Aguilar-Hernandez N, Meyer L, Lopez S, DuBois RM, Arias CF. Protein Disulfide Isomerase A4 Is Involved in Genome Uncoating during Human Astrovirus Cell Entry. Viruses. 2020;13(1). Epub 20201231. doi: 10.3390/v13010053. PubMed PMID: 33396308; PubMed Central PMCID: PMCPMC7824429.

9. Sidik SM, Huet D, Ganesan SM, Huynh MH, Wang T, Nasamu AS, et al. A Genome-wide CRISPR Screen in Toxoplasma Identifies Essential Apicomplexan Genes. Cell. 2016;166(6):1423-35 e12. Epub 20160902. doi: 10.1016/j.cell.2016.08.019. PubMed PMID: 27594426; PubMed Central PMCID: PMCPMC5017925.

10. Barylyuk K, Koreny L, Ke H, Butterworth S, Crook OM, Lassadi I, et al. A Comprehensive Subcellular Atlas of the Toxoplasma Proteome via hyperLOPIT Provides Spatial Context for Protein Functions. Cell Host Microbe. 2020;28(5):752-66 e9. Epub 20201013. doi: 10.1016/j.chom.2020.09.011. PubMed PMID: 33053376; PubMed Central PMCID: PMCPMC7670262.

11. Mazzarella RA, Srinivasan M, Haugejorden SM, Green M. ERp72, an abundant luminal endoplasmic reticulum protein, contains three copies of the active site sequences of protein disulfide isomerase. J Biol Chem. 1990;265(2):1094-101. PubMed PMID: 2295602.

12. Hayano T, Kikuchi M. Cloning and sequencing of the cDNA encoding human P5. Gene. 1995;164(2):377-8. doi: 10.1016/0378-1119(95)00474-k. PubMed PMID: 7590364.

13. Lay AJ, Dupuy A, Hagimola L, Tieng J, Larance M, Zhang Y, et al. Endoplasmic reticulum protein 5 attenuates platelet endoplasmic reticulum stress and secretion in a mouse model. Blood Adv. 2023;7(9):1650-65. doi: 10.1182/bloodadvances.2022008457. PubMed PMID: 36508284; PubMed Central PMCID: PMCPMC10182305.
